# Characterization of Nigerian breast cancer reveals prevalent homologous recombination deficiency and aggressive molecular features

**DOI:** 10.1038/s41467-018-06616-0

**Published:** 2018-10-16

**Authors:** Jason J. Pitt, Markus Riester, Yonglan Zheng, Toshio F. Yoshimatsu, Ayodele Sanni, Olayiwola Oluwasola, Artur Veloso, Emma Labrot, Shengfeng Wang, Abayomi Odetunde, Adeyinka Ademola, Babajide Okedere, Scott Mahan, Rebecca Leary, Maura Macomber, Mustapha Ajani, Ryan S. Johnson, Dominic Fitzgerald, A. Jason Grundstad, Jigyasa H. Tuteja, Galina Khramtsova, Jing Zhang, Elisabeth Sveen, Bryce Hwang, Wendy Clayton, Chibuzor Nkwodimmah, Bisola Famooto, Esther Obasi, Victor Aderoju, Mobolaji Oludara, Folusho Omodele, Odunayo Akinyele, Adewunmi Adeoye, Temidayo Ogundiran, Chinedum Babalola, Kenzie MacIsaac, Abiodun Popoola, Michael P. Morrissey, Lin S. Chen, Jiebiao Wang, Christopher O. Olopade, Adeyinka G. Falusi, Wendy Winckler, Kerstin Haase, Peter Van Loo, John Obafunwa, Dimitris Papoutsakis, Oladosu Ojengbede, Barbara Weber, Nasiru Ibrahim, Kevin P. White, Dezheng Huo, Olufunmilayo I. Olopade, Jordi Barretina

**Affiliations:** 1 Institute for Genomics and Systems Biology, University of Chicago, Chicago, IL 60637 USA; 2 Novartis Institutes for BioMedical Research, Cambridge, MA 02139 USA; 3 Center for Clinical Cancer Genetics & Global Health, Department of Medicine, University of Chicago, Chicago, IL 60637 USA; 4 Department of Pathology and Forensic Medicine, Lagos State University Teaching Hospital, Ikeja, Lagos Nigeria; 5 Department of Pathology, University of Ibadan, Ibadan, Oyo Nigeria; 6 Department of Epidemiology and Biostatistics, School of Public Health, Peking University Health Science Center, Beijing, 100191 China; 7 Institute for Advanced Medical Research and Training, College of Medicine, University of Ibadan, Ibadan, Oyo Nigeria; 8 Department of Surgery, University of Ibadan, Ibadan, Oyo Nigeria; 9 Department of Surgery, Lagos State University Teaching Hospital, Ikeja, Lagos Nigeria; 10 Department of Pharmaceutical Chemistry, University of Ibadan, Ibadan, Oyo Nigeria; 11 Oncology Unit, Department of Radiology, Lagos State University, Ikeja, Lagos Nigeria; 12 Department of Public Health Sciences, University of Chicago, Chicago, IL 60637 USA; 13 The Francis Crick Institute, 1 Midland Road, London, NW1 1AT UK; 14 Department of Human Genetics, University of Leuven, Oude Markt 13, Leuven, 3000 Belgium; 15 Centre for Population and Reproductive Health, College of Medicine, University of Ibadan, Ibadan, Oyo Nigeria; 16 Tempus Labs Inc., Chicago, IL USA; 17 Present Address: Cancer Science Institute of Singapore, National University of Singapore, 14 Medical Drive, Singapore, 117599 Singapore; 18 Present Address: Girona Biomedical Research Institute (IDIBGI), Girona, 17007 Spain

## Abstract

Racial/ethnic disparities in breast cancer mortality continue to widen but genomic studies rarely interrogate breast cancer in diverse populations. Through genome, exome, and RNA sequencing, we examined the molecular features of breast cancers using 194 patients from Nigeria and 1037 patients from The Cancer Genome Atlas (TCGA). Relative to Black and White cohorts in TCGA, Nigerian HR + /HER2 − tumors are characterized by increased homologous recombination deficiency signature, pervasive *TP53* mutations, and greater structural variation—indicating aggressive biology. *GATA3* mutations are also more frequent in Nigerians regardless of subtype. Higher proportions of APOBEC-mediated substitutions strongly associate with *PIK3CA* and *CDH1* mutations, which are underrepresented in Nigerians and Blacks. *PLK2*, *KDM6A*, and *B2M* are also identified as previously unreported significantly mutated genes in breast cancer. This dataset provides novel insights into potential molecular mechanisms underlying outcome disparities and lay a foundation for deployment of precision therapeutics in underserved populations.

## Abstract (web summary)

Research on racial and ethnic influence on breast cancer mortality is stymied by a lack of genomic studies in diverse populations. Here, the authors genomically interrogate 194 Nigerian breast cancers, unveiling molecular features that could explain the high mortality rate from breast cancer in an indigenous African population.

## Introduction

Breast cancer is a heterogeneous disease comprising distinct subtypes. Both global burden and severity of the disease vary widely across populations, with women of African ancestry being diagnosed at a younger age, having more clinically aggressive disease and advanced stage at diagnosis, as well as having higher mortality rates than age-matched women of European or Asian ancestry^1–4^. Molecular and genetic characteristics strongly influence breast cancer prognosis and treatment, with HER2 amplification (human epidermal growth factor receptor 2 [ERBB2]) and hormone receptor (HR; estrogen receptor [ER] and progesterone receptor [PR]) expression being the best examples.

Recent large sequencing studies, for instance the International Cancer Genome Consortium (ICGC) and The Cancer Genome Atlas (TCGA), have refined our knowledge of the genomic landscape and pathogenesis of breast cancer, have provided insight into tumor evolution and mechanisms of drug resistance, and have laid a pathway to deployment of precision therapeutics^5–15^. Moreover, these large public datasets have also enhanced our understanding on the divergent mutation accretion processes; most notably in breast cancer, studies have shown high APOBEC (apolipoprotein B mRNA editing enzyme, catalytic polypeptide-like)-related mutagenesis, especially in HER2 + tumors^16^, whereas *BRCA1/2* mutations are strongly associated with signatures depicting DNA repair deficiency^17^.

The cases used to elucidate the genetic basis of breast cancer have been overwhelmingly from women of European ancestry, which reiterates the need for data from underrepresented ethnicities^18–20^. Moreover, paucity of data from African countries potentially widens the knowledge gap that contributes to global disparities in breast cancer outcomes. To get a comprehensive understanding of the genetic architecture of breast cancer in West Africans, the founder population of a large proportion of Black women in the United States, we conducted whole-genome sequencing (WGS), whole-exome sequencing (WES), and transcriptome sequencing (RNA sequencing (RNA-seq)) on 194 tumors from Nigerian patients and performed a comparative analysis with Black women of African ancestry and White women of European ancestry from the United States in TCGA. In comparison with the TCGA cohorts, we observe that HR + /HER2 − Nigerians are enriched for molecular characteristics associated with aggressive biology. To the best of our knowledge, combined with African American patients in TCGA, this is the largest breast cancer genomics study on tumors from women of African ancestry to date.

## Results

### Study populations

The Nigerian cohort comprised 194 breast cancer patients: 40 with WGS data, 129 with WES data, and 103 with RNA-seq data (Supplementary Fig. 1). Of the 1097 TCGA breast cancer patients with either WES (*n* = 1035) or WGS (*n* = 84), 1030 were assigned without ambiguity to 3 ancestral race groups (Black, ≥ 50% African; White, ≥ 90% European; Asian, ≥ 90% Asian ancestry) and the other 67 had mixed racial background (Supplementary Data 1a–e). DNA sequencing data from all samples was uniformly processed using the SwiftSeq workflow (manuscript in preparation). Patient clinical and pathologic characteristics are shown in Supplementary Tables 1–5. Nigerians were much younger and had more advanced stage at diagnosis than patients in the TCGA cohort, reflecting population structure and lack of screening in the country.

### Mutation landscape of Nigerians compared with Americans

**Fig. 1:**
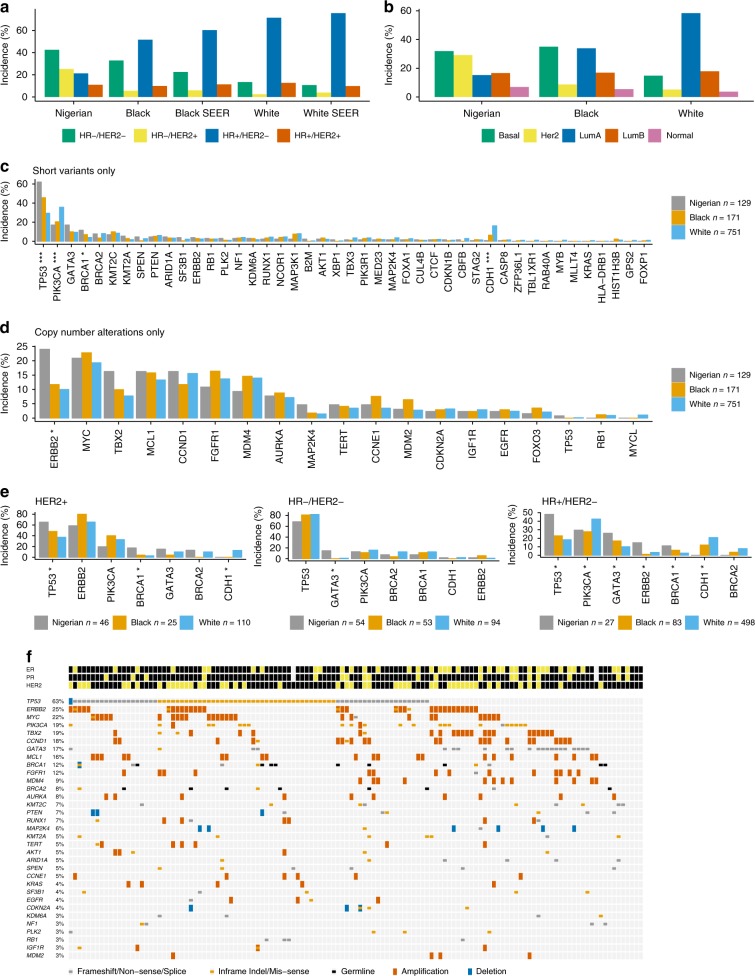
Landscape of breast cancer in Nigerians compared to Black and White Americans. **a** Proportion of IHC subtypes in the Nigerian, Black, and White cohorts from TCGA and in the SEER database. **b** Proportion of PAM50 subtypes in Nigerians, Blacks, and Whites. **c** Comparison of the frequencies of short variants (SNVs and indels) in 44 breast cancer drivers in all cohorts. **d** Alteration frequencies of 19 genes recurrently affected by CNAs (homozygous deletions and amplifications). **e** Comparison of key breast cancer drivers stratified by IHC subtype. Both short variants and copy number events are included. **f** Oncoprint of short mutations and CNAs in Nigerians. Recurrently mutated genes that were altered least 3% of Nigerians are shown. *P < 0.05; **P < 0.001; ***P < 0.0001 (Fisher’s exact with *P*-values adjusted via the Benjamini–Hochberg method)

Congruous with previous studies including the Surveillance Epidemiology End Results dataset^2,21^, we observed a strong enrichment of HR −/HER2 − (i.e., triple-negative for ER/PR/HER2; 43% in Nigerian vs. 33% in Black and 13% in White) and HR −/HER2 + (25 vs. 6 and 2%) subtypes in the Nigerian cohort (Fig. 1a). PAM50 subtyping revealed a similar enrichment of Basal-like (32 vs. 35 and 15%) and HER2 enriched (29 vs. 9 and 5%) in Nigerian women (Fig. 1b).

Across all 1164 individuals—both TCGA and Nigerians—with WES data, we identified 25 genes that were significantly mutated above background (MutSigCV, *Q* < 0.05). Three of these genes (*PLK2*, *KDM6A*, and *B2M*; Supplementary Methods; Supplementary Fig. 2) had little or no previous evidence of harboring mutations that drive breast carcinogenesis. A fourth gene, *GPS2*, was also identified by Bailey et al.^22^ while this manuscript was under review. Notably, mutations in *PLK2* (Fisher’s exact, *P* = 0.05) and *KDM6A* (*P* = 0.06) were enriched within HER2 + patients. Combined with previously reported significantly mutated genes in breast cancer^13,23^, this resulted in 44 driver genes. These genes, along with those recurrently affected by copy number changes^6^ (Supplementary Table 6), were used for gene-centric comparisons by race/ethnicity.

Consistent with the aggressive subtype composition in Nigerians, we found an enrichment of *TP53* alterations (62 vs. 46 and 29%; Fisher’s exact, Benjamini–Hochberg [BH] *P* < 0.0001) as well as a lower prevalence of *PIK3CA* mutations (17 vs. 20 and 36%; BH *P* < 0.0001) (Fig. 1c). Combined *BRCA1* germline and somatic variants were also enriched in the Nigerian cohort (11.6 vs. 7.0 and 4.0%; BH *P* = 0.03). *CDH1* mutation was rare in Nigerians (0.8 vs. 6.4 and 16.2%; BH *P* < 0.0001), whereas *GATA3* alterations were more common in this population (17.1 vs. 10.0% and 9.5%; BH *P* = 0.24).

When comparing recurrently gained or lost regions as identified by GISTIC2 (Supplementary Fig. 3; Supplementary Methods), we found that all high confidence peaks identified in the Nigerian cohort had corresponding peaks within 10 Mb in the combined TCGA cohort. In line with immunohistochemistry (IHC) and PAM50, the *ERBB2* locus (17q12) was enriched in Nigerians (amplified in 24 vs. 12 and 10%; BH *P* = 0.002), as was its wide neighboring peak at 17q23.1 (*TBX2* locus, BH *P* = 0.1) (Fig. 1d).

Within IHC subtypes, significantly mutated genes and copy number peaks generally displayed similar proportions across ethnicities (Fig. 1e, f), suggesting that most mutation frequency differences reflects subtype differences across ethnicities. Within the HR + /HER2 − subtype, however, there were more *TP53* and *GATA3* mutations, and fewer *PIK3CA* and *CDH1* mutations in Nigerians, compared with TCGA Blacks and Whites (all *P* < 0.05). These results are not strongly influenced by age (Supplementary Methods) and suggest that HR + /HER2 − breast cancers in Nigerian women have genomic lesions consistent with more aggressive disease.

### Mutation signatures across subtypes and driver mutations

**Fig. 2:**
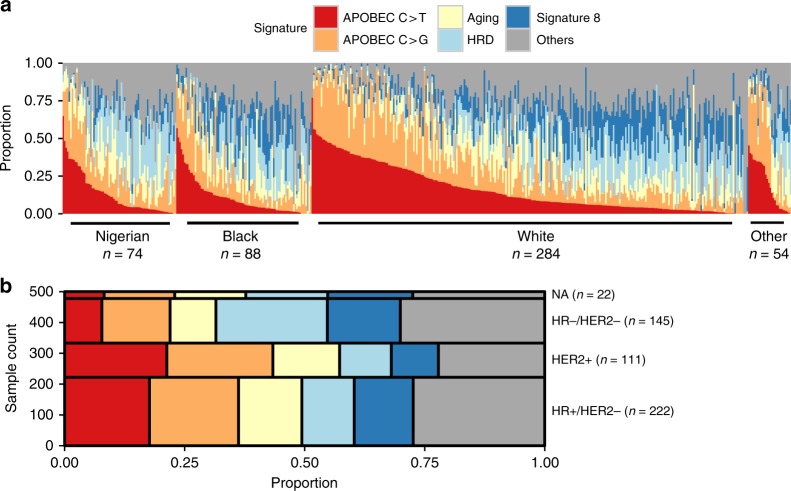
Mutation signature contributions across race/ethnicity and subtype. **a** The contribution (proportion) of mutation signatures (Signatures D, E, F, and G are combined into “Other”) within each individual. Individuals are partitioned by race/ethnicity and ordered by APOBEC C > T signature contributions (high to low). The number of individuals representing each cohort is shown. **b** Mekko plot of the proportional contributions of mutation signatures across IHC subtypes

We next extracted breast cancer mutational signatures in the 122 WGS and 500 WES samples from Nigerian and TCGA cohorts harboring 100 or more mutations (Supplementary Methods). Of the nine independently identified signatures, signatures A (APOBEC C > T), B (APOBEC C > G), C (Aging), H (Signature 8), and I (homologous recombination deficiency [HRD]) closely matched to previously identified breast cancer signatures (Supplementary Figs. 4 and 5A). Given that these five signatures had high correlation between exomes and genomes (Supplementary Fig. 5b), we examined those in subsequent analyses. Combined, they explain the vast majority of mutations regardless of race/ethnicity (Fig. 2a) or subtype (Fig. 2b).

We observed increased contributions from APOBEC C > T (Mann–Whitney *U* [MWU], *P* = 3.5 × 10^−9^) and APOBEC C > G (*P* = 0.044) signatures in HR + tumors compared with HR − tumors, which is consistent with previous findings^24,25^. On average, the HRD signature was more active in HR − tumors (*P* = 2.2 × 10^−15^) (Supplementary Fig. 6a–d). Consistent with previous work^16^, HER2 + tumors had the highest contributions from APOBEC C > T and C > G signatures (*P* = 1.6 × 10^−8^ and *P* = 9.1 × 10^−4^, respectively) (Fig. 2b; Supplementary Fig. 6e, f). Similarly, we recapitulated the known aging signature associations (Supplementary Methods) and confirmed higher HRD contributions in individuals harboring deleterious germline or somatic *BRCA1/2* mutations (*P* = 6.2 × 10^−7^)^17^.

**Fig. 3:**
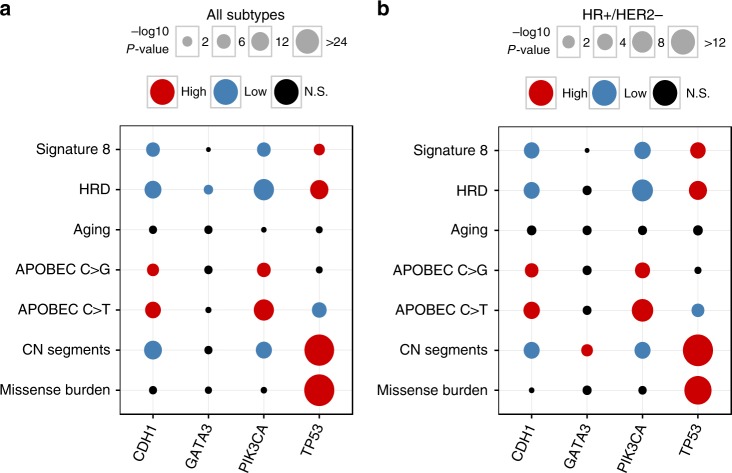
Associations between genome-wide oncogenic features and the mutation status of common driver genes. Dot plot depicting the relationships between mutation status in *TP53*, *PIK3CA*, *CDH1*, and *GATA3*, and mutation signatures (APOBEC C > T, APOBEC C > G, aging, HRD, and signature 8), missense mutation burden, and copy number (CN) segments **a** across all IHC subtypes (*n* = 500) and **b** within HR +/HER2 − (*n* = 222). Only TCGA data, including samples lacking mutation signature estimates, was used for CN associations (all subtype *n* = 1,023; HR +/HER2 − *n* = 635). No samples were excluded based on race/ethnicity. Comparisons between mutation status and genomic features were performed with Mann–Whitney *U* and *P*-values were corrected for multiple testing (Benjamini–Hochberg method). Circle size is proportional to the magnitude of the − log10 BH *P*-value (i.e., lower BH *P*-values have larger circles). If mutation status associated with a significant increase or decrease of a genomic feature, the corresponding circle is colored red or blue, respectively. Non-significant (NS) comparisons are colored black

*TP53* mutations were associated with higher HRD contributions (MWU, *P* = 2.1 × 10^−13^), higher missense mutation burden (*P* = 6.5 × 10^−45^), and increased copy number segmentation (*P* = 2.0 × 10^−43^) (Fig. 3a; Supplementary Methods). In contrast, *CDH1* or *PIK3CA* mutations—which frequently co-occur (*P* = 3.8 × 10^−8^)—were associated with lower HRD contributions (*CDH1 P* = 5.2 × 10^−11^; *PIK3CA P* = 2.1 × 10^−17^) in addition to higher contributions from APOBEC C > T (*P* = 3.0 × 10^−9^; *P* = 3.8 × 10^−17^) and C > G (*P* = 1.7 × 10^−4^; *P* = 2.1 × 10^−6^) (Fig. 3a). Importantly, these significant associations persisted even when considering only HR + /HER2 − tumors (Fig. 3b). These findings suggest a consistent interplay between driver mutations and the relative activity of mutational processes.

### Mutation signatures across race and ethnicity

**Fig. 4:**
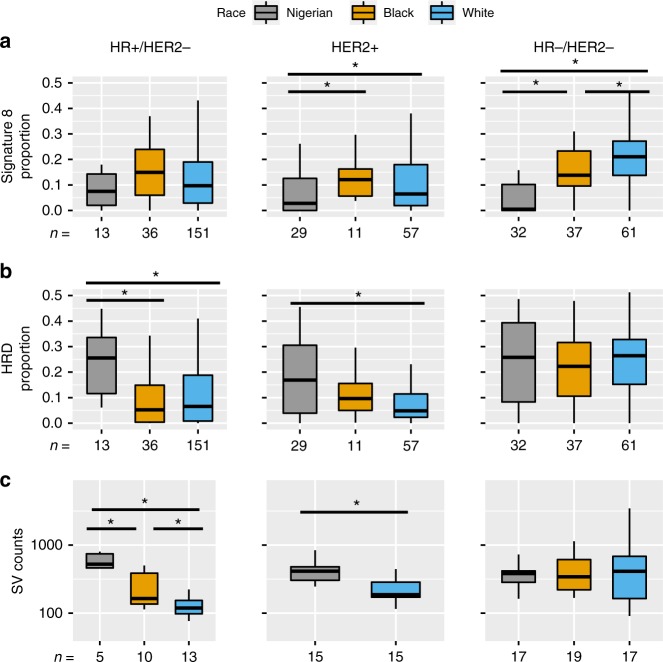
Mutation signature contributions and structural variant counts by race/ethnicity and IHC subtype. Mutation signature contributions from **a** signature 8 and **b** HRD subdivided by race/ethnicity and IHC subtype. **c** Boxplots representing the number of SVs identified across WGS samples partitioned by race/ethnicity and IHC subtype. Asterisks denote significant differences (*P* < 0.05) between groups using Kruskal–Wallis tests followed by post-hoc comparisons with Dunn’s test. Each box represents the upper and lower quartiles of the data, and the median is depicted with a horizontal line. Upper and lower whiskers extend to the largest and smallest values within [1.5 × interquartile range], respectively

Signature 8 demonstrated substantial contribution differences between cohorts. This effect was the most pronounced in HR −/HER2 − tumors, where Nigerians and Blacks (*P* = 4.4 × 10^−6^), Nigerians and Whites (*P* = 4.6 × 10^−12^), as well as Blacks and Whites (*P* = 0.023) were significantly different from one another (Fig. 4a). Notably, Whites presented with remarkably higher signature 8 in HR −/HER2 − (mean = 20.6%) compared with HR + /HER2 − (mean = 12.2%) tumors (*P* = 3.4 × 10^−7^), which was recapitulated using WGS data (*P* = 6.9 × 10^−3^) (Supplementary Fig. 8a, b). These subtype differences were not observed for either Nigerians or Blacks.

In the HR + /HER2 − subtype, the APOBEC C > T signature displayed differences by race/ethnicity with Nigerian and Black cohorts having lower APOBEC C > T contributions compared with Whites (MWU, *P* < 0.05). In the HR −/HER2 − subtype, Nigerians had increased APOBEC C > G signature relative to the Black and White cohorts (*P* < 0.05) (Supplementary Fig. 7a, b). Strikingly, HR +/HER2 − Nigerian tumors had higher HRD signature contributions compared with both Black (*P* = 1.8 × 10^−4^) and White (*P* = 1.6 × 10^−4^) cohorts (Fig. 4b). This finding was confirmed using data from WGS (Supplementary Fig. 8c). Structural variants (SVs) are more prevalent in tumor types with HRD defects such as ovarian and basal-like breast cancers^13,26^. In this same set of genomes, Nigerians had more SVs than both Black (MWU, *P* = 0.03) and White cohorts (*P* = 2.8 × 10^−4^). Similar with the HRD signature, SVs counts in HR +/HER2 − Nigerians (~551 SVs per genome) were reminiscent of HR −/HER2 − (~626 SVs per genome) (Fig. 4c). Differences between Nigerians and Whites in HRD signature and SVs (both *P* < 2.0 × 10^−3^) extended to HER2 + cases as well (Fig. 4b, c). Taken together, multiple lines of evidence suggest that HR +/HER2 − Nigerians have increased HRD and genomic complexity compared with the Black and White cohorts. Furthermore, genome data suggests a potentially more granular stratification by African ancestry.

We postulated that increased HRD in HR +/HER2 − Nigerians may be explained by increased prevalence of *TP53* mutations as well as fewer *PIK3CA* and *CDH1* mutations—although not necessarily causatively. Using multivariate modeling (Supplementary Methods), we investigated the effect of race/ethnicity on HRD adjusting for age and missense burden, as well as mutation status in *TP53*, *BRCA1/2*, *PIK3CA*, and *CDH1*. Although many of these factors have significant, independent effects, they cannot entirely account for the racial/ethnic HRD disparities seen across HR +/HER2 − tumors.

### HRD-APOBEC signature balance

**Fig. 5:**
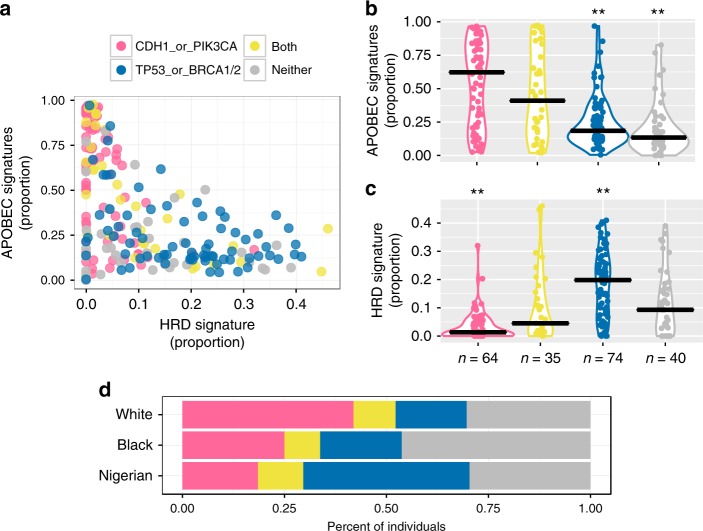
Driver gene mutations associate with APOBEC and HRD signature balance in HR+/HER2- breast cancer. **a** For each tumor, the proportion of APOBEC signatures (sum of APOBEC C > T and C > G) by the proportion of HRD is shown. Each patient is colored based on harboring a *CDH1* or *PIK3CA* mutation (pink), a *TP53* or *BRCA1/2* (including germline) mutation (blue), mutations from both aforementioned categories (yellow), or mutations in neither of the aforementioned categories (gray). These values are decomposed into violin plots for **b** APOBEC and **c** HRD signatures, respectively. Horizontal black bars represent the median contribution proportion for each group. Between group comparisons were made using a Kruskal–Wallis test followed by Dunn’s test. Panels **a**–**c** were not restricted by race/ethnicity. **d** The proportion of HR +/HER2 − individuals falling into each mutational group by race/ethnicity (*n* White = 465; *n* Black = 80; *n* Nigerian = 27). This also includes samples for which mutation signatures were not estimated. **Groups that were significantly different (*P* < 0.05) from all three other categories

Several threads of evidence suggest a possible interplay between the HRD and APOBEC signature contributions, particularly in HR +/HER2 − breast cancers: (1) we identified racial/ethnic differences in mutation prevalence for *TP53*, *CDH1*, and *PIK3CA*; (2) we found associations between these mutations and mutation signatures (Fig. 3a); and (3) consistent with differential mutation status, HRD activity was increased in Nigerians, whereas APOBEC C > T displayed reduced activity in Nigerians and Blacks compared with Whites (Supplementary Fig. 7a). Furthermore, within this subtype, HRD had a notable negative correlation with both the APOBEC C > T (*ρ* = − 0.56, permutation test *P* < 0.0001; Supplementary Methods) and APOBEC C > G (*ρ* = − 0.30, *P* < 0.0001) signatures. Integrating these findings, we postulated that a balance of HRD and APOBEC signature contributions exists and can be discriminated—if not dictated—by mutations in *BRCA1/2* (germline and somatic), *TP53*, *PIK3CA*, and *CDH1*. For each tumor, we combined APOBEC C > T and C > G contributions, and plotted them against that of HRD (Fig. 5a). Tumors were partitioned based on the presence of *CDH1* or *PIK3CA* mutations (*CDH1/PIK3CA*), *TP53* or *BRCA1/2* mutations (*TP53/BRCA1/BRCA2*), mutations from both aforementioned categories (Both), or mutations in neither of the aforementioned categories (Neither). APOBEC contributions were significantly higher in *CDH1/PIK3CA* compared with the *TP53/BRCA1/BRCA2* (Dunn’s test, *P* = 1.8 × 10^−6^) and Neither (*P* = 7.2 × 10^−9^) groups. Tumors harboring mutations from both groups (Both) had lower APOBEC contributions than *CDH1/PIK3CA* (*P* = 0.11), yet higher than *TP53/BRCA1/BRCA2* (*P* = 5.0 × 10^−3^) (Fig. 5b). In contrast, *TP53/BRCA1/BRCA2* had significantly higher HRD contributions than all other groups (*P CDH1/PIK3CA* = 9.9 × 10^−15^; Both = 1.6 × 10^−4^; Neither = 2.1 × 10^−4^), whereas *CDH1/PIK3CA* had significantly lower contributions than all other groups (*P* Both = 3.5 × 10^−3^; Neither = 1.2 × 10^−3^) (Fig. 5c). These findings were similar when considering all samples simultaneously (Supplementary Fig. 9a–c).

The signature patterns for the Neither group most closely resembled those of *TP53/BRCA1/BRCA2* (Fig. 5a–c), suggesting that there may be other mechanisms, such as inactivation of other homologous recombination genes^27^ or *BRCA1/2* methylation^28^, which promote increased HRD activity. When looking at the proportion of these mutational groups across HR +/HER2 − samples (including those without signature estimates), the groups with the highest HRD and lowest APOBEC—*TP53/BRCA1/BRCA2* and Neither—encompassed 70.3% Nigerians and 66.3% Blacks but only 47.7% of Whites (*χ*^2^-test, *P* = 1.2 × 10^−3^) (Fig. 5d). This suggests that individuals with African ancestry are more likely to fall within mutational groups associated with increased HRD and lower APOBEC contributions. Consistent with this assertion, the HR +/HER2 − Black cohort had greater copy number segmentation (MWU, *P* = 0.022), more structural variation (Dunn’s test, *P* = 0.028), and increased HRD in WGS (Dunn’s test, *P* = 0.015) compared with Whites (Fig. 4b; Supplementary Fig. 8c). Throughout African ancestry tumors, prevalent aggressive and limited favorable molecular features could in part explain known racial/ethnic mortality disparities within the HR +/HER2 − subtype^29^. This has significant clinical implications, because HRD tumors are more likely to be sensitive to platinum-based chemotherapy, PARP (poly (ADP-ribose) polymerase) inhibition, and immunotherapy^28^.

### Infiltrating immune cell inference by RNA signatures

**Fig. 6:**
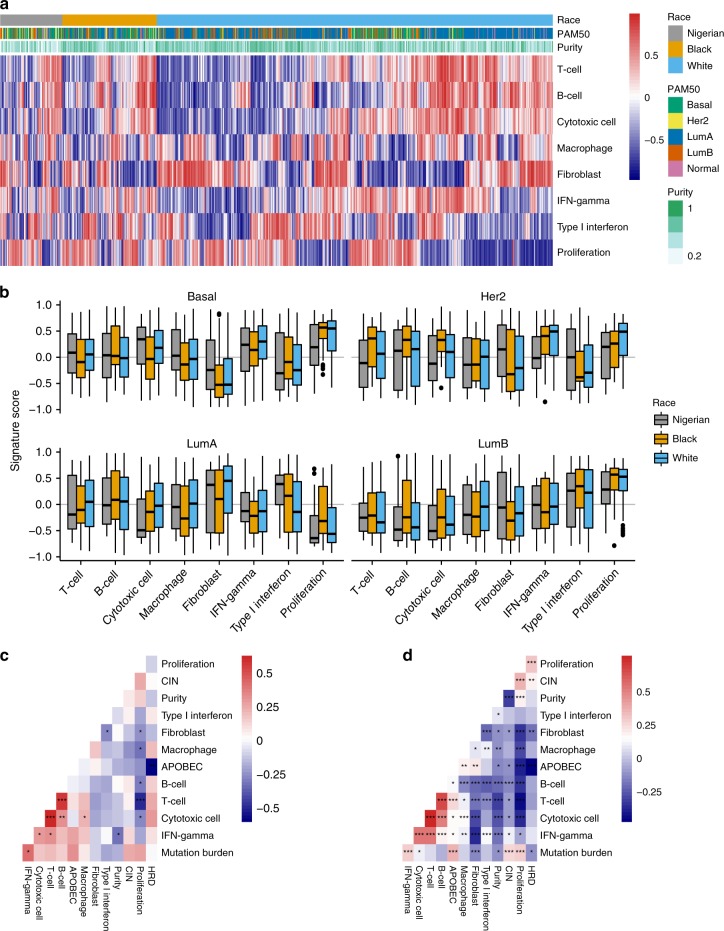
Gene signatures of immune cell infiltration. **a** Heatmap visualizing gene signature activation in all 1040 patients with RNA-seq data (Nigerian *n* = 103, Black *n* = 183, and White *n* = 754). High signature scores (red) indicate high overall expression of genes in the signatures, whereas low values (blue) indicate low expression. **b** Distribution of signature scores across PAM50 subtypes and ethnicities. **c**, **d** Pairwise Pearson’s correlation of immune signatures as well as potential predictors of response to immunotherapy (APOBEC, HRD, CIN, mutation burden). The Nigerian data are shown in **c** and the combined Black and White cohorts in **d**. CIN chromosomal instability; HRD homologous recombination deficiency; IFN interferon. Each box represents the upper and lower quartiles of the data and the median is depicted with a horizontal line. Upper and lower whiskers extend to largest and smallest values within [1.5 × interquartile range], respectively. *P < 0.05; **P < 0.001, ***P < 0.0001 (all adjusted using the Benjamini–Hochberg method)

Given the high HRD signature activity and the fact that DNA repair gene alterations have been linked to checkpoint inhibitor efficacy, we next investigated gene expression signatures related to immune cell infiltration, or immune signatures, with RNA-seq (Fig. 6a and Supplementary Table 7). Most immune signatures—B-cell, Cytotoxic T cell, Fibroblast, Interferon (IFN)-γ, Type I IFN, and Proliferation—displayed statistically significant differences across PAM50 subtypes (analysis of variance, all *P* < 0.0001; Supplementary Methods). Racial differences adjusted for PAM50 subtype, however, were modest (Fig. 6b and Supplementary Fig. 1,0). The Cytotoxic cell signature (*P* = 0.004) was lower in Nigerians in all subtypes but Basal, whereas the Fibroblast signature (*P* = 0.01) was consistently highest in Nigerians. Type I IFN signature scores (*P* = 0.01) were enriched in Luminal subtypes for both Nigerians and Blacks, which potentially indicates that tumors from these racial groups would respond better to immunotherapy^30^. Lastly, macrophage infiltration in Nigerians was highest in the Basal subtype, similar to what has been reported in other studies, including one in a small subset of Nigerian patients^31,32^.

We next tested these immune signatures for association with potential predictors of response to immunotherapy. We considered the combined APOBEC C > T and C > G, and the HRD mutation signatures as the two independent mutational processes generating putative neoantigens, as well as mutation burden and chromosomal instability (CIN)^33–35^. APOBEC mutation signature contribution was positively correlated with mutation burden (*ρ* = 0.35, Spearman’s rank correlation, BH *P* < 0.0001). Consistent with recent reports, we found APOBEC contribution being further associated with increased T-cell infiltration (*ρ* = 0.25, BH *P* < 0.0001) and CIN being positively correlated with mutation burden (*ρ* = 0.28, BH *P* < 0.0001), while negatively correlated with T-cell infiltration (*ρ* = − 0.08, BH *P* < 0.01)^34,35^. The same trends were observed in the Nigerian and TCGA cohorts separately with similar effect sizes (Fig. 6c, d), although, in the former, most were not significant after multiple testing correction potentially due to the smaller sample size (Fig. 6a).

## Discussion

To date, this study is the largest genomic analysis of breast cancer among women of African ancestry. Aggressive molecular subtypes were found to be more prevalent in Nigerian patients, which has been consistently documented in breast tumors across West Africa^2^. The extent to which this disparity represents disparate biology, environmental influences, or a combination thereof remains unknown. Recently, ER expression was demonstrated to be a heritable trait in breast cancer^36^, suggesting that genetically influenced basal expression levels may contribute to subtype differentiation. Given that genetic background associates with phenotypes relevant to breast cancer, it is reasonable to postulate that patterns of somatic mutations may differ across genetically distinct populations. Here we have shown that regardless of subtype, aggressive molecular features are prevalent in breast tumors from Nigerian women.

Including Nigerian samples along with TCGA allowed us to identify *PLK2*, *KDM6A*, and *B2M* as novel significantly mutated genes in breast cancer, with the former two enriched in the HER2 + subtype. *PLK2* is a cell cycle regulator and presumed tumor suppressor, whereas *KDM6A* is a chromatin modifier frequently mutated in other cancer types (e.g., pancreatic, esophageal, and bladder)^37–40^. *B2M* inactivation was recently reported to be a recurrent event in lung cancer and potentially affects response to anti-PD-1/anti-PD-L1 therapies^41^. Further studies to characterize the role for these genes in HER2 + tumors specifically and breast cancer in general are warranted.

The mutational landscape and signature patterns differed across racial/ethnic populations. In particular, the relatively younger Nigerian patients had more *TP53* and *GATA3* mutations than Blacks in TCGA, whereas both African ancestry groups had higher prevalence of these mutations than Whites. The frequencies of prognostically favorable *PIK3CA* and *CDH1* mutations were lower in women of African ancestry than in Whites, which may reflect differences in breast cancer risk factors across populations. Even when restricting to ER +/HER2 − breast cancer, tumors from Nigerian women were characterized by canonically aggressive molecular features, such as higher contributions from the HRD mutational signature, *TP53* mutations, and increased structural variation. Along with more pervasive HR negativity and HER2 positivity, the aggressive features of HR + tumors provide biological insight to why breast cancers in the unscreened and relatively younger female populations of West Africa are often fatal^42^. This study lays the foundation for a more concerted effort to reduce global disparities in cancer outcomes by first closing the knowledge gaps. Given the genomic landscape, Nigerian women would benefit from increased access to genomically tailored clinical trials and more effective treatments such as HER2-targeted therapy and PARP inhibition for HER2 + and HRD-deficient tumors, respectively^28^.

There are certain limitations to this study including the relatively small sample size of Nigerian tumors and the fact that both TCGA and this study used convenient samples ascertained in Hospitals and may not reflect population rates. Nonetheless, this study underscores the need to include diverse populations when identifying and pursuing novel therapeutic targets^18^. It is possible that genetic and environmental factors not only drive subtype differentiation but also dictate evolutionary dynamics of a tumor. This latter assertion could help explain the observed mutational differences between racial/ethnic groups, a pattern which has also been noted comparing Black and Whites with colorectal cancer in the United States^43^. Similarly, strong associations between driver mutations and mutation signature contributions (e.g., *PIK3CA* and APOBEC signatures) pose a causality dilemma suited for further biological and epidemiological investigations. Overall, our results justify the need for future studies integrating germline and somatic genetics, as well as environmental factors, in order to better understand the root causes of disparities in breast cancer outcomes and develop more effective interventions to achieve health equity.

## Methods

### Biospecimen collection and pathological assessment

This study was embedded within the Nigerian Breast Cancer Study (NBCS) and approved by the Institutional Review Board of all participating institutions. Patient ascertainment and details of the study have been previously published^2,44,45^. In collaboration with Novartis, NBCS was extended to Lagos State University Teaching Hospital (LASUTH). A grand total of 493 subjects were recruited from University College Hospital, Ibadan (UCH; *n* = 284) and LASUTH (*n* = 209) between February 2013 and September 2015. Each patient gave written informed consent before participation in the study. Six biopsy cores and peripheral blood were collected from each patient. Two biopsy cores were used for routine formalin fixation for clinical diagnosis and the remaining four cores were preserved in PAXgene Tissue containers (Qiagen, CA) for subsequent genomic material extraction. In addition, 27 mastectomy tissues were preserved in RNAlater. Complete pathology assessment was done central by study pathologists. Tumor burden was assessed based on cellularity, histology type, and morphological quality of tissue using TCGA best practices, and only tissues containing 60% or more tumor cellularity were used for WGS. For WES, tissues containing 30% or more tumor cellularity were used. IHC on ER, PR, and HER2 were performed centrally in Nigeria and further reviewed in the United States. Cases with discordant results were again reviewed and resolved by the study pathologists. IHC scoring variables for Allred scoring algorithm were captured according to the 2013 ASCO/CAP standard reporting guidelines. Briefly, for ER and PR testing, immunoreactive tumor cells < 1% was recorded as negative and those with ≥ 1% were reported positive. All the positive ER and PR cases were graded in percentages stained cells and further scored in line with the Allred scoring system. Percentage of tumor staining for HER2 test were also reported along with a score of 0 and 1 + as negative, 2 + as equivocal, and 3 + as positive case. In addition, genomic copy number calls of HER2 and chromosome 17 ploidy were used as alternative to HER2 fluorescent in situ hybridization test. Overall, IHC calls were corroborated *ESR1*, *PGR*, and *ERBB2* expression using RNA-seq (Supplementary Fig. 1,1a–c).

### Sample selection and genomic material extraction

Breast tumors were selected for sequencing following the TCGA guidelines^6^. Tumor samples containing > 60% tumor cellularity were selected for DNA extraction using PAXgene Tissue DNA kit (Qiagen). Gentra Puregene Blood Kit (Qiagen) was used to extract genomic DNA from blood. Extracted DNA were quality controlled for its purity, quantity, and integrity. Identity of the extracted DNA were tested using AmpFlSTR Identifiler PCR Amplification Kit (Thermo Fisher Scientific). Samples that match > 80% of the short tandem repeat profiles between tumor and germline DNA were considered authentic. RNA was extracted from PAXgene fixed tissues using the PAXgene Tissue RNA kit (Qiagen). RNA integrity (RIN) was determined for all samples by the RIN score given by the TapeStation (Agilent) read out. RNA samples that had RIN scores of 4 and above were included in downstream sequencing analysis.

### Next-generation sequencing data generation

WES and RNA-seq were carried out at the Novartis Next Generation Diagnostics facility. Exome enrichment was performed on libraries (prepared by Illumina TruSeq Nano DNA Library Prep Kit) passing QC using Agilent SureSelect XT Human All Exon V4 baits and SureSelect XT capture enrichment reagents. Passing captured libraries are combined in equimolar pools with other captured libraries of compatible adapter barcodes. These pools were normalized with concentration and were sequenced on the Illumina HiSeq 2500 sequencer. Tumor samples had an average coverage depth of 139 × (63 × to 265 ×), normals 52 × (19 ×–205 ×; Supplementary Data 2a). WGS was performed at the University of Chicago High-throughput Genome Analysis Core (HGAC) and at the New York Genome Center (NYGC). Libraries were prepared using the Illumina Truseq DNA PCR-free Library Preparation Kit. Libraries were sequenced on an Illumina HiSeq 2000 sequencer at HGAC using 2 × 100bp paired-end format and HiSeq × sequencer (v2.5 chemistry) at NYGC using 2 × 150 bp cycles. Mean coverage depth tumor was at 98.5 × and normal was at 34.2 × (Supplementary Data 2b). For RNA-seq, total RNA were constructed into poly-A selected Illumina-compatible cDNA libraries using the Illumina TruSeq RNA Sample Prep kit. Passing cDNA libraries were combined in equimolar pools with other libraries of compatible adapter barcodes and later sequenced on the Illumina HiSeq 2500 sequencer. Average number of mapped reads per sample was 97 million (ranging from 36 to 232 million).

### Alignment of DNA sequence to reference genome

For both exomes and genomes, reads were aligned to GRCh37 from GATK data bundle version 2.8 ([https://software.broadinstitute.org/gatk/]) using BWA-MEM (v0.7.12; [http://bio-bwa.sourceforge.net/])^46^. Duplicate reads were removed using PicardTools MarkDuplicates (v1.119; [https://broadinstitute.github.io/picard/]). Using a custom Fluidigm SNP panel, we confirmed that whole-exome BAM files matched the library DNA, to identify sample swaps in the sequencing lab or bioinformatics pipelines.

### Calling somatic single nucleotide variants

Single-nucleotide variants (SNVs) were called using both MuTect (v1.1.7; [http://archive.broadinstitute.org/cancer/cga/mutect])^47^ and Strelka (v1.0.13; [https://sites.google.com/site/strelkasomaticvariantcaller/])^48^ with default parameters, except Strelka’s depth filter was not used for exomes (isSkipDepthFilters = 1). Variants were called on the entirety of the genome in order to detect and retain any high-quality off-target calls. Any variant call that did not meet ‘PASS’ criteria for either algorithm was discarded. For a given tumor-normal pair, only SNVs called by both MuTect and Strelka were retained. Furthermore, using 1088 blood germline exomes (959 TCGA BRCA; 129 Nigerian), we constructed a panel of normal samples. For a given normal sample, a site needed to be covered by a minimum of ten reads to be included. Any SNV that was supported by 5% or more of reads (MAPQ (MAPping Quality) ≥ 20; Base quality ≥ 20) in two or more samples was removed. SNVs were later annotated with Oncotator ([http://archive.broadinstitute.org/cancer/cga/oncotator])^49^ and those that met the required criteria ("COSMIC_n_overlapping_mutation > 1" AND "1000gp3_AF ≤ 0.005" AND "ExAC_AF ≤ 0.005") were considered likely to be somatic and were retained. This panel of normal process was also repeated for genomes (normal sample *n* = 124). All subsequent SNV calls were annotated by Variant Effect Predictor (VEP) (v79; [http://useast.ensembl.org/info/docs/tools/vep/index.html])^50^.

### Calling somatic insertions and deletions (indels)

Small indels were called using Scalpel (v0.5.3; [http://scalpel.sourceforge.net/]) in somatic mode^51,52^. Variants were only called in known genic regions as defined by Broad.human.exome.b37.interval.bed from the GATK data bundle version 2.8. To minimize the number of false-positive calls, we employed the two-pass option. Default Scalpel filters were implemented, which required a minimum alternative allele count of four in the tumor, no alternative allele present in the normal, and a minimum tumor variant allele frequency of 5%. In addition, indel calls located in repetitive genomic regions (via DustMasker; [https://www.ncbi.nlm.nih.gov/IEB/ToolBox/CPP_DOC/lxr/source/src/app/dustmasker/]) or found in the 1000 Genomes Project Phase 3 release ([http://www.internationalgenome.org/]) were removed^53,54^. Finally, we implemented a pseudo panel of normals by aggregating all putative indel calls that failed Scalpel filters due to ‘HighVafNormal’ or ‘HighAltCountNormal’. Any indel that failed in two or more samples was filtered. The remaining calls were annotated using VEP.

### Calling germline SNVs and indels

For both exomes and genomes, reads were aligned to GRCh37 from GATK data bundle version 2.8. Duplicate reads were removed using PicardTools MarkDuplicates (v1.119). Both SNVs and indels were called using Platypus (v0.7.9.1; [http://www.well.ox.ac.uk/platypus]) in single-sample mode^55^. Only variants passing the Platypus ‘PASS’ filter were considered for downstream analysis. The resulting set of variants were annotated using VEP. All variants with an ExAC^56^ allele frequency ≥ 0.05 were discarded. Remaining variants were considered deleterious if they were annotated as HIGH impact by VEP or a missense variant with a CADD^57^ score > 25.

### Calling copy number alterations

Allele-specific copy number in whole-exome data was called using PureCN (v1.7.16; [http://bioconductor.org/packages/PureCN/])^58^. Alternative purity and ploidy solutions were considered (Supplementary Fig. 3a–d). Genes were called amplified if the median exon copy number was ≥ 6 for focal gains ( < 3 Mb) or ≥ 7 for non-focal gains. Genes with median exon copy number of 0 were called lost. Non-focal amplifications of tumor suppressor genes were excluded^59^. As Affymetrix Genome-Wide Human SNP Array 6.0 data were available for the TCGA cohort, copy number calling was performed using ASCAT ([https://www.crick.ac.uk/peter-van-loo/software/ASCAT]; Supplementary Methods). Amplifications and deletions were called exactly as in the exome data. GISTIC (v2.0.22; [http://archive.broadinstitute.org/cancer/cga/gistic])^60^ was used to identify significantly gained or lost genomic regions in the Nigerian cohort. TCGA GISTIC2 results were obtained from the BROAD FireBrowse portal ([http://firebrowse.org/]). CIN was defined as the fraction of the genome with copy number alteration. Details are provided in the Supplementary Methods.

### Calling SVs in WGS

SVs (deletions, duplications, and inversions) were called with both Delly (v0.6.1; [https://github.com/dellytools/delly]^61^ and Lumpy Express (v0.2.10; [https://github.com/arq5x/lumpy-sv])^62^. A panel of normal samples was constructed by taking all Delly SVs calls made in at least one (*n* = 124) normal sample, regardless of “PASS” or “LowQual” in the FILTER field. Any SV found within the panel of normals was removed from the analysis. All Delly SVs passing the aforementioned filters were queried within the matched Lumpy calls. Delly SVs corroborated by a Lumpy call (same SV type and breakpoints within 500 bp [up or downstream]) were retained. These consensus SVs were filtered if a breakpoint (from either Delly or Lumpy) fell within a repetitive genomic region according to DustMasker. Lastly, inversions were required to have split read evidence (at least one read) from both Delly and Lumpy.

### Estimating genetic ancestry of study population

We estimated the ancestry of breast cancer patients from TCGA using principal component analysis as practiced by TCGA Analysis Working Group^36^. According to the estimated proportion of ancestry, patients were grouped into genomic Black ( ≥ 50% African ancestry), genomic White ( ≥ 90% European ancestry), and genomic Asian ( ≥ 90% Asian ancestry). All Nigerian patients were assumed to be 100% African with little to no admixture with other populations^63^.

### Significantly mutated genes

To detect significantly mutated genes we used MutSigCV (v1.4; [http://software.broadinstitute.org/cancer/software/genepattern/modules/docs/MutSigCV])^23,64^. SNV and indel variant call formats from 1164 individuals were annotated with Oncotator using the oncotator_v1_ds_Jan262014 database. MutSigCV was then invoked with default parameters on the Oncotator generated MAF file. To reduce common false positives, we allowed only a single non-silent indel within a given gene per sample. Finally, for any gene to be called significantly mutated, we required it to have more than two individuals harboring non-silent mutations across the entire dataset.

### Mutation signatures in WES and WGS

The Bioconductor ([https://bioconductor.org/]) package SomaticSignatures ([https://bioconductor.org/packages/SomaticSignatures/])^65^ was used to estimate somatic mutational signatures. The ability to reliably call mutation signatures depends on sufficient numbers of mutations. To this point, we used all high-quality exome SNVs, regardless of whether they are coding or non-coding. Any sample containing at least 100 SNVs was included for downstream assessment. In addition, in order to stimulate more accurate signature estimates, 122 WGS tumor-normal pairs were also included in addition to 500 WES pairs (Supplementary Data 1c). To account for variable mutation counts across samples, we used SomaticSignatures to normalize the mutation matrix before performing non-negative matrix factorization. We elected to estimate 9 signatures (Supplementary Fig. 4), as that was (1) consistent with the number of signatures identified previously in breast cancer ([http://cancer.sanger.ac.uk/cosmic/signatures]) and (2) as 9 signatures explained ~99% of variance when using 122 genomes alone. Using matrix algebra on the resulting exposure and mutation matrices, we calculated the relative contribution of the nine signatures on each sample. Contributions represent the proportion of mutations assigned to given mutation signature within each tumor (Supplementary Methods). Exomes were used for all mutation signature analyses unless explicitly stated.

### RNA-seq analysis and immune signatures

Gene expression measurements were uniformly calculated using Omicsoft ArraySuite® software ([http://www.omicsoft.com/array-studio])^66^ for Nigerian and TCGA samples. The RNA-seq reads passing quality control were aligned to the Human B37 genome. Read counts for the UCSC gene models were calculated by the software. The gene counts were upper quartile normalized with the edgeR Bioconductor/R package ([https://bioconductor.org/packages/edgeR/])^67^ and batch normalized using ComBat as implemented in the sva package ([https://bioconductor.org/packages/sva/])^68^. Transcripts per million expression values were calculated based on the normalized counts. PAM50 classification was carried out using the pbcmc package ([https://bioconductor.org/packages/pbcmc/])^69^ using the robust parameter. Nigerian PAM50 classifications were consistent with IHC calls (Supplementary Fig. 1,2). To characterize the immune and stromal microenvironment of these tumors, we assessed the expression of several pre-specified sets of immune and stromal cell gene expression markers (Supplementary Table 7). Gene signature scores were calculated using the GSVA R/Bioconductor package ([https://www.bioconductor.org/packages/GSVA/])^70^.

### Statistical methods

All statistical calculations were completed in in R. Names of the performed tests are provided in the text and all *P*-values are two-sided. Non-parametric tests were used when the underlying data types often lacked normality (e.g., mutation signature contributions). All boxplots throughout the manuscript are Tukey’s style.

### Code availability

SwiftSeq is available at [https://github.com/PittGenomics/SwiftSeq]

### URLs

COSMiC, http://cancer.sanger.ac.uk/cosmic; Gene Ontology Consortium, [http://www.geneontology.org/]; ICGC, [https://www.genome.gov/10001688/]; SwiftSeq, [https://github.com/PittGenomics/SwiftSeq]; TCGA, [https://cancergenome.nih.gov/]

## Electronic supplementary material

Supplementary Information

Peer Review File

Description of Additional Supplementary Files

Supplementary Data 1

Supplementary Data 2

## Data Availability

Raw TCGA data used in this analysis were downloaded from TCGA Data Portal or Cancer Genomics Hub, and their UUIDs are listed in Supplementary Data 1f–h. Access to the harmonized variant calls that support the findings of this study are available on request from the corresponding author (O.I.O). The raw sequencing data from Nigerian cases is available through dbGaP under Study Accession phs001687.v1.p1.

## References

[1] Torre LA (2015). Global cancer statistics, 2012. CA Cancer J. Clin..

[2] Huo D (2009). Population differences in breast cancer: survey in indigenous African women reveals over-representation of triple-negative breast cancer. J. Clin. Oncol..

[3] Servick K (2014). Breast cancer. Breast Cancer.

[4] Newman LA (2002). African-American ethnicity, socioeconomic status, and breast cancer survival: a meta-analysis of 14 studies involving over 10,000 African-American and 40,000 White American patients with carcinoma of the breast. Cancer.

[5] Lehmann BD (2011). Identification of human triple-negative breast cancer subtypes and preclinical models for selection of targeted therapies. J. Clin. Invest..

[6] Cancer Genome Atlas Network (2012). Comprehensive molecular portraits of human breast tumours. Nature.

[7] Stephens PJ (2012). The landscape of cancer genes and mutational processes in breast cancer. Nature.

[8] Banerji S (2012). Sequence analysis of mutations and translocations across breast cancer subtypes. Nature.

[9] Curtis C (2012). The genomic and transcriptomic architecture of 2,000 breast tumours reveals novel subgroups. Nature.

[10] Shah SP (2012). The clonal and mutational evolution spectrum of primary triple-negative breast cancers. Nature.

[11] Wang Y (2014). Clonal evolution in breast cancer revealed by single nucleus genome sequencing. Nature.

[12] Pereira B (2016). The somatic mutation profiles of 2,433 breast cancers refines their genomic and transcriptomic landscapes. Nat. Commun..

[13] Nik-Zainal S (2016). Landscape of somatic mutations in 560 breast cancer whole-genome sequences. Nature.

[14] Morganella S (2016). The topography of mutational processes in breast cancer genomes. Nat. Commun..

[15] Berger AC (2018). A Comprehensive Pan-Cancer Molecular Study of gynecologic and breast cancers. Cancer Cell.

[16] Roberts SA (2013). An APOBEC cytidine deaminase mutagenesis pattern is widespread in human cancers. Nat. Genet..

[17] Alexandrov LB (2013). Signatures of mutational processes in human cancer. Nature.

[18] Spratt DE (2016). Racial/ethnic disparities in genomic sequencing. JAMA Oncol..

[19] Bustamante CD, Burchard EG, De la Vega FM (2011). Genomics for the world. Nature.

[20] Popejoy AB, Fullerton SM (2016). Genomics is failing on diversity. Nature.

[21] Eng A, McCormack V, dos-Santos-Silva I (2014). Receptor-defined subtypes of breast cancer in indigenous populations in Africa: a systematic review and meta-analysis. PLoS Med..

[22] Bailey MH (2018). Comprehensive characterization of cancer driver genes and mutations. Cell.

[23] Lawrence MS (2013). Mutational heterogeneity in cancer and the search for new cancer-associated genes. Nature.

[24] Periyasamy M (2015). APOBEC3B-mediated cytidine deamination is required for estrogen receptor action in breast cancer. Cell Rep..

[25] Zhang Y, Delahanty R, Guo X, Zheng W, Long J (2015). Integrative genomic analysis reveals functional diversification of APOBEC gene family in breast cancer. Hum. Genom..

[26] Patch AM (2015). Whole-genome characterization of chemoresistant ovarian cancer. Nature.

[27] Polak P (2017). A mutational signature reveals alterations underlying deficient homologous recombination repair in breast cancer. Nat. Genet..

[28] Davies H (2017). HRDetect is a predictor of BRCA1 and BRCA2 deficiency based on mutational signatures. Nat. Med..

[29] O’Brien KM (2010). Intrinsic breast tumor subtypes, race, and long-term survival in the Carolina Breast Cancer Study. Clin. Cancer Res..

[30] Sistigu A (2014). Cancer cell-autonomous contribution of type I interferon signaling to the efficacy of chemotherapy. Nat. Med..

[31] Campbell MJ (2011). Proliferating macrophages associated with high grade, hormone receptor negative breast cancer and poor clinical outcome. Breast Cancer Res. Treat..

[32] Adisa CA (2012). Biology of breast cancer in Nigerian women: a pilot study. Ann. Afr. Med..

[33] Rizvi NA (2015). Cancer immunology. Mutational landscape determines sensitivity to PD-1 blockade in non-small cell lung cancer. Science.

[34] Smid M (2016). Breast cancer genome and transcriptome integration implicates specific mutational signatures with immune cell infiltration. Nat. Commun..

[35] Davoli, T., Uno, H., Wooten, E. C. & Elledge, S. J. Tumor aneuploidy correlates with markers of immune evasion and with reduced response to immunotherapy. *Science* **355**, pii: eaaf8399 (2017). 10.1126/science.aaf8399 PMC5592794 28104840

[36] Huo D (2017). Comparison of breast cancer molecular features and survival by African and European ancestry in The Cancer Genome Atlas. JAMA Oncol..

[37] Bailey P (2016). Genomic analyses identify molecular subtypes of pancreatic cancer. Nature.

[38] Gao YB (2014). Genetic landscape of esophageal squamous cell carcinoma. Nat. Genet..

[39] Gui Y (2011). Frequent mutations of chromatin remodeling genes in transitional cell carcinoma of the bladder. Nat. Genet..

[40] Villegas E (2014). Plk2 regulates mitotic spindle orientation and mammary gland development. Development.

[41] Pereira, C. et al. Genomic profiling of patient-derived xenografts for lung cancer identifies B2m inactivation impairing immunorecognition. *Clin. Cancer Res*. 23, 3203–3213 (2016). 10.1158/1078-0432.CCR-16-1946-T 10.1158/1078-0432.CCR-16-1946 28302866

[42] Vanderpuye V (2017). An update on the management of breast cancer in Africa. Infect. Agent. Cancer.

[43] Guda K (2015). Novel recurrently mutated genes in African American colon cancers. Proc. Natl Acad. Sci. USA.

[44] Adebamowo CA (2003). Obesity and height in urban Nigerian women with breast cancer. Ann. Epidemiol..

[45] Huo D (2008). Parity and breastfeeding are protective against breast cancer in Nigerian women. Br. J. Cancer.

[46] Li, H. Aligning sequence reads, clone sequences and assembly contigs with BWA-MEM. Preprint at https://arxiv.org/abs/1303.3997/ (2013).

[47] Cibulskis K (2013). Sensitive detection of somatic point mutations in impure and heterogeneous cancer samples. Nat. Biotechnol..

[48] Saunders CT (2012). Strelka: accurate somatic small-variant calling from sequenced tumor-normal sample pairs. Bioinformatics.

[49] Ramos AH (2015). Oncotator: cancer variant annotation tool. Hum. Mutat..

[50] McLaren W (2016). The Ensembl variant effect predictor. Genome Biol..

[51] Narzisi G (2014). Accurate de novo and transmitted indel detection in exome-capture data using microassembly. Nat. Methods.

[52] Fang H (2014). Reducing INDEL calling errors in whole genome and exome sequencing data. Genome Med..

[53] Morgulis A, Gertz EM, Schaffer AA, Agarwala R (2006). A fast and symmetric DUST implementation to mask low-complexity DNA sequences. J. Comput. Biol..

[54] 1000 Genomes Project Consortium (2015). A global reference for human genetic variation. Nature.

[55] Rimmer A (2014). Integrating mapping-, assembly- and haplotype-based approaches for calling variants in clinical sequencing applications. Nat. Genet..

[56] Lek M (2016). Analysis of protein-coding genetic variation in 60,706 humans. Nature.

[57] Kircher M (2014). A general framework for estimating the relative pathogenicity of human genetic variants. Nat. Genet..

[58] Riester M (2016). PureCN: copy number calling and SNV classification using targeted short read sequencing. Source Code Biol. Med..

[59] Futreal PA (2004). A census of human cancer genes. Nat. Rev. Cancer.

[60] Mermel CH (2011). GISTIC2.0 facilitates sensitive and confident localization of the targets of focal somatic copy-number alteration in human cancers. Genome Biol..

[61] Rausch T (2012). DELLY: structural variant discovery by integrated paired-end and split-read analysis. Bioinformatics.

[62] Layer RM, Chiang C, Quinlan AR, Hall IM (2014). LUMPY: a probabilistic framework for structural variant discovery. Genome Biol..

[63] Huo D (2012). Evaluation of 19 susceptibility loci of breast cancer in women of African ancestry. Carcinogenesis.

[64] Lawrence MS (2014). Discovery and saturation analysis of cancer genes across 21 tumour types. Nature.

[65] Gehring JS, Fischer B, Lawrence M, Huber W (2015). SomaticSignatures: inferring mutational signatures from single-nucleotide variants. Bioinformatics.

[66] Hu J, Ge H, Newman M, Liu K (2012). OSA: a fast and accurate alignment tool for RNA-Seq. Bioinformatics.

[67] Robinson MD, McCarthy DJ, Smyth GK (2010). edgeR: a Bioconductor package for differential expression analysis of digital gene expression data. Bioinformatics.

[68] Leek JT, Johnson WE, Parker HS, Jaffe AE, Storey JD (2012). The sva package for removing batch effects and other unwanted variation in high-throughput experiments. Bioinformatics.

[69] Fresno C (2017). A novel non-parametric method for uncertainty evaluation of correlation-based molecular signatures: its application on PAM50 algorithm. Bioinformatics.

[70] Hanzelmann S, Castelo R, Guinney J (2013). GSVA: gene set variation analysis for microarray and RNA-seq data. BMC Bioinformatics.

