# Author Correction: Characterization of Nigerian breast cancer reveals prevalent homologous recombination deficiency and aggressive molecular features

**DOI:** 10.1038/s41467-018-07886-4

**Published:** 2019-01-14

**Authors:** Jason J. Pitt, Markus Riester, Yonglan Zheng, Toshio F. Yoshimatsu, Ayodele Sanni, Olayiwola Oluwasola, Artur Veloso, Emma Labrot, Shengfeng Wang, Abayomi Odetunde, Adeyinka Ademola, Babajide Okedere, Scott Mahan, Rebecca Leary, Maura Macomber, Mustapha Ajani, Ryan S. Johnson, Dominic Fitzgerald, A. Jason Grundstad, Jigyasa H. Tuteja, Galina Khramtsova, Jing Zhang, Elisabeth Sveen, Bryce Hwang, Wendy Clayton, Chibuzor Nkwodimmah, Bisola Famooto, Esther Obasi, Victor Aderoju, Mobolaji Oludara, Folusho Omodele, Odunayo Akinyele, Adewunmi Adeoye, Temidayo Ogundiran, Chinedum Babalola, Kenzie MacIsaac, Abiodun Popoola, Michael P. Morrissey, Lin S. Chen, Jiebiao Wang, Christopher O. Olopade, Adeyinka G. Falusi, Wendy Winckler, Kerstin Haase, Peter Van Loo, John Obafunwa, Dimitris Papoutsakis, Oladosu Ojengbede, Barbara Weber, Nasiru Ibrahim, Kevin P. White, Dezheng Huo, Olufunmilayo I. Olopade, Jordi Barretina

**Affiliations:** 1 Institute for Genomics and Systems Biology, University of Chicago, Chicago, IL 60637 USA; 2 Novartis Institutes for BioMedical Research, Cambridge, MA 02139 USA; 3 Center for Clinical Cancer Genetics & Global Health, Department of Medicine, University of Chicago, Chicago, IL 60637 USA; 4 Department of Pathology and Forensic Medicine, Lagos State University Teaching Hospital, Ikeja, Lagos Nigeria; 5 Department of Pathology, University of Ibadan, Ibadan, Oyo Nigeria; 6 Department of Epidemiology and Biostatistics, School of Public Health, Peking University Health Science Center, Beijing 100191, China; 7 Institute for Advanced Medical Research and Training, College of Medicine, University of Ibadan, Ibadan, Oyo Nigeria; 8 Department of Surgery, University of Ibadan, Ibadan, Oyo Nigeria; 9 Department of Surgery, Lagos State University Teaching Hospital, Ikeja, Lagos Nigeria; 10 Department of Pharmaceutical Chemistry, University of Ibadan, Ibadan, Oyo Nigeria; 11 Oncology Unit, Department of Radiology, Lagos State University, Ikeja, Lagos Nigeria; 12 Department of Public Health Sciences, University of Chicago, Chicago, IL 60637 USA; 13 The Francis Crick Institute, 1 Midland Road, London NW1 1AT, UK; 14 Department of Human Genetics, University of Leuven, Oude Markt 13, Leuven 3000, Belgium; 15 Centre for Population and Reproductive Health, College of Medicine, University of Ibadan, Ibadan, Oyo Nigeria; 16 Present Address: Tempus Labs, Inc., Chicago, IL USA; 17 Present Address: Cancer Science Institute of Singapore, National University of Singapore, 14 Medical Drive, Singapore 117599, Singapore; 18 Girona Biomedical Research Institute (IDIBGI), 17007 Girona 17007, Spain

Correction to: *Nature Communications* 10.1038/s41467-018-06616-0, published online: 16 Oct 2018

The original version of this Article contained an error in the author affiliations.

The affiliation of Kevin P. White with Tempus Labs, Inc. Chicago, IL, USA, was inadvertently omitted.

This has now been corrected in both the PDF and HTML versions of the Article.

