# Supplementary material for: Characterization of Nigerian breast cancer reveals prevalent homologous recombination deficiency and aggressive molecular features
Source: Nat Commun. 2018 Oct 16;9:4181. doi: 10.1038/s41467-018-06616-0 (PMC6191428; doi:10.1038/s41467-018-06616-0)
Supplement: Supplementary file 1 — Supplementary Information [file 41467_2018_6616_MOESM1_ESM.pdf]

Supplementary Information

# Characterization of Nigerian Breast Cancer Reveals High Rates of Homologous Recombination Deficiency

Pitt & Riester *et al*.

# Supplementary Methods

## Novel significantly mutated genes in breast cancer

*PLK2* (polo-like kinase 2) and *KDM6A* (lysine demethylase 6A) had non-silent mutation occurrence similar to other known drivers at 1.4% (n = 16) and 2.1% (n = 24), respectively. For samples with additional WGS data, all mutations within these genes were validated (Supplementary Table 8).

## Whole-exome copy number calling

The required coverages of targeted exons and 200kb off-target bins were calculated and GC-bias corrected using PureCN. To maximize the number of heterozygous SNPs informative for allele-specific copy number estimation, we included all variants in the 50 base pair flanking regions of targets. Position-specific mapping bias estimates of known germline SNPs were obtained by providing PureCN a variant call format (VCF) file containing variants present in 5 or more of the normal samples. Since accurate copy number calling is notoriously difficult in low purity exome data, we compared the PureCN (v1.7.16)[1] calls with 2 other recently published tools, FACETS (v0.5.6)[2] and Sequenza (v2.1.2)[3]. Cases for which the PureCN estimates of tumor ploidy or purity differed by 0.5 or 0.1, respectively, from the median of the estimates from the 3 tools were manually curated. PureCN further flags samples for manual curation and all flagged samples were curated. A comparison of the un-curated calls is shown in Supplementary Fig. 3a-b. We ran all tools as recommended in the corresponding documentations. In total, 3.9% of samples showed discordant purity estimates. PureCN is the only tool that includes somatic point mutations in the purity estimation and in half of the discordant samples, PureCN correctly identified a very low tumor contribution as revealed by low allelic fractions of somatic SNVs. Ploidy was as expected more discordant, with 7.8% of samples showing a difference in ploidy of 0.5 or higher. Discordant samples had low purity (average 37% vs. 47% for concordant samples; two-sided t-test $P$ = 0.05). For 2% of samples we changed the ploidy estimate. The remaining samples were either of too low purity or quality to confidently call ploidy (i.e. genome doubling yes vs. no) or the PureCN estimates were more plausible.

## Recurrent copy number alterations

GISTIC2.0[4] was run with parameters -ta 0.3 -td -0.3 -conf 0.9 -broad 1 -brlen 0.5. 0.3 approximately matched the average log-ratio standard deviation in normal samples. We excluded GISTIC peaks (Supplementary Fig. 3e-f) for which the majority of exons displayed a high variance in tumor vs. normal coverage log-ratios in the pool of normal samples. The affected deletion peaks in large repetitive regions were 2q32, 4q13.3, 7q21.11, 10p11.22 and Xq22.3. Furthermore, only homozygous deletions were counted (i.e. single copy losses were not included in Fig. 1 of the main text).

## Affymetrix SNP6 array copy number calling

Copy number changes across TCGA breast cancer tumors were called using the ASCAT algorithm[5]. Initially, CEL files provided by TCGA Data Portal, for both malignant and normal tissue, were processed using PennCNV[6] to obtain logR and BAF data. Since samples profiled via SNP arrays are prone to wave artifacts, the logR was subsequently corrected for GC content. Copy number profiles for all tumor samples were inferred using the ASCAT computational framework (version 2.4.2) from the BAF and corrected LogR data. Only copy

number profiles for samples that had matching exome data were used for analysis (Supplementary Data 1d)

## Adjusting for age in mutation proportion comparisons

Within HR+/HER2-, we explored racial differences in mutation proportions after adjusting for age using logistic regression. There was a significant difference in proportions of *TP53* mutation across racial cohorts, mainly between Nigerians and the two other groups ($P < 0.001$). Similarly, there is significant difference in proportion of *PIK3CA* mutation across racial cohorts. The difference exists between Whites and other two African ancestry groups, though the difference between Whites and Nigerians alone was not statistically significant. Age-adjustment did not account for racial differences in *GATA3* mutation proportions in Nigerians ($P = 0.05$). Lastly, there is a higher proportion of *CDH1* mutation in Whites than in Nigerians. Because the contingency table contained a '0' cell, multi-variable logistic regression cannot be easily fit. However, since the difference was large (0 vs. 21%), it cannot be explained by age alone. Of note, Nigerians had only 1 lobular case (Supplementary Table 1)

## Mutation signatures

**Signature training.** We utilized the SomaticSignatures[7] to jointly identify mutation signatures and estimate their contributions to individual tumors. To help ensure robust signature determination, we included data from 82 TCGA and 40 Nigerian whole genome tumor-normal pairs 82. Across exomes with ≥ 100 SNVs (n = 500) and 122 genomes (Supplementary Data 1c), we estimated nine signatures, A through I (Supplementary Fig. 4**)**. For all analyses we used percentage of mutations assigned to each signature (i.e., contribution) rather than the total number of mutations attributed to a signature. This distinction is important as high APOBEC contributions, for example, do not necessarily imply APOBEC hypermutation. 59 individuals had both somatic WES and WGS data. For all nine signatures, we examined the correlation of contributions between exomes and genomes. Signatures A (Rho = 0.87), B (0.92), C (0.69), H (0.86), and I (0.75) all exhibited strong correlation (Rho ≥ ~0.7) (Supplementary Fig. 5b). APOBEC C>T (Signature A) and C>G (Signature B) contributions were highly correlated (Rho = 0.65, Spearman's rank correlation, $P < 2.2 \times 10^{-16}$). Contributions from the aging signature (Signature C) we also positively correlated with age at diagnosis (Rho = 0.18, $P = 7.3 \times 10^{-5}$).

**Comparison with reported signatures.** We compared our mutation signature matrices with the 30 previously reported signatures downloaded from the Catalog of Somatic Mutation in Cancer (COSMiC), containing previously identified 30 signatures operative across a variety of cancer types. We used the Kullback-Leibler Divergence[8] to compare our signatures to those from COSMiC:

$$KL(P,Q)=\frac{1}{2}\left(\sum_{i=1}^{96} P_i \ln\frac{P_i}{Q_i}+\sum_{i=1}^{96} Q_i \ln\frac{Q_i}{P_i}\right)$$

With this approach, we determined the best representing known COSMiC signature for each of the signature we identified (Supplementary Fig. 5a). Signatures A (COSMiC signature 2;

APOBEC C>T), B (COSMiC signature 13; APOBEC C>G), C (COSMiC signature 1; Aging), H (COSMiC signature 3; homologous recombination deficiency [HRD]), and I (COSMiC signature 8; Unknown etiology) all closely matched to signatures known to be operative in breast cancer (Fig. 2A).

**Signature permutations.** For each individual, we randomly selected two signature contribution values without replacement. These values were assigned to dummy signature 1 and dummy signature 2, respectively. Dummy signatures were subsequently correlated using Spearman's method. This process was repeated 10,000 times to construct a null Rho distribution. Permuted P-values were calculated by comparing empirical Rho values to the null distribution.

## Immune Signatures

**Testing for association with PAM50 subtypes and race.** ANOVA and median regression with bootstrapping standard errors was used to test for association of immune signature scores (Supplementary Data 1e) with PAM50 subtypes and race. The median regression is robust to the skewness of the distribution in immune signatures. We fit a model with interaction terms for race and subtypes and found none of the interactions to be significant. Therefore, the analysis using all subjects provided more power and could be justified over subgroup analyses. In addition, PCA analysis (Supplementary Fig. 10) showed that subtype, not race was the most significant source of variance.

## Supplementary Table 1. Summary statistics for WES, WGS, and RNA-seq samples

| | Whole exome sequencing (WES, N=1,164) | | | | | Whole genome sequencing (WGS, N=124 ) | | | | | RNA sequencing (RNA, 1040) | | |
|---|---|---|---|---|---|---|---|---|---|---|---|---|---|
| | TCGA White | TCGA Black | TCGA Asian | TCGA Unassigned | Nigerians | TCGA White | TCGA Black | TCGA Asian | TCGA Unassigned | Nigerians | TCGA White | TCGA Black | Nigerians |
| Number | 751 | 171 | 54 | 59 | 129 | 46 | 30 | 3 | 5 | 40 | 754 | 183 | 103 |
| Age | 59.5±13.0 | 56.0±13.2 | 55.0±12.2 | 55.9±14.2 | 49.7±12.4 | 60.2±13.3 | 57.0±14.1 | 65.7±13.1 | 45.6±13.4 | 52.6±13.3 | 58.6±12.9 | 56.3±13.3 | 51.6±12.4 |
| ER | | | | | | | | | | | | | |
| + | 580 (77.2) | 102 (59.7) | 31 (57.4) | 44 (74.6) | 38 (29.5) | 25 (54.4) | 9 (30.0) | 1 (33.3) | 4 (80.0) | 9 (22.5) | 574 (76.1) | 110 (60.1) | 31 (30.1) |
| - | 128 (17.0) | 65 (38.0) | 19 (35.2) | 14 (23.7) | 88 (68.2) | 20 (43.5) | 21 (70.0) | 2 (66.7) | 1 (20.0) | 31 (77.5) | 137 (18.2) | 69 (37.7) | 67 (65.1) |
| Unknown | 43 (5.7) | 4 (2.3) | 4 (7.4) | 1 (1.7) | 3 (2.3) | 1 (2.2) | 0 (0.0) | 0 (0.0) | 0 (0.0) | 0 (0.0) | 43 (5.7) | 4 (2.2) | 5 (4.8) |
| PR | | | | | | | | | | | | | |
| + | 511 (68.0) | 86 (50.3) | 28 (51.9) | 35 (59.3) | 19 (14.5) | 19 (41.3) | 8 (26.7) | 1 (33.3) | 4 (80.0) | 3 (7.5) | 501 (66.4) | 90 (49.2) | 15 (14.6) |
| - | 195 (26.0) | 81 (47.4) | 22 (40.7) | 23 (39.0) | 107 (83.0) | 27 (58.7) | 22 (73.3) | 2 (66.7) | 1 (20.0) | 34 (85.0) | 207 (27.5) | 89 (48.6) | 83 (80.6) |
| Unknown | 45 (6.0) | 4 (2.3) | 4 (7.4) | 1 (1.7) | 3 (2.3) | 0 (0.0) | 0 (0.0) | 0 (0.0) | 0 (0.0) | 3 (7.5) | 46 (6.1) | 4 (2.2) | 5 (4.8) |
| HER2 | | | | | | | | | | | | | |
| + | 110 (14.7) | 25 (14.6) | 17 (31.5) | 10 (17.0) | 45 (34.9) | 15 (32.6) | 0 (0.0) | 2 (66.7) | 2 (40.0) | 16 (40.0) | 93 (12.3) | 27 (14.8) | 44 (42.7) |
| - | 623 (83.0) | 140 (81.9) | 35 (64.8) | 48 (81.4) | 83 (64.3) | 30 (65.2) | 29 (96.7) | 0 (0.0) | 3 (60.0) | 24 (60.0) | 641 (85.0) | 150 (82.0) | 52 (50.5) |
| Unknown | 18 (2.4) | 6 (3.5) | 2 (3.7) | 1 (1.7) | 1 (0.8) | 1 (2.2) | 1 (3.3) | 1 (33.3) | 0 (0.0) | 0 (0.0) | 20 (2.7) | 6 (3.3) | 4 (3.9) |
| Equivocal | 0 (0.0) | 0 (0.0) | 0 (0.0) | 0 (0.0) | 0 (0.0) | 0 (0.0) | 0 (0.0) | 0 (0.0) | 0 (0.0) | 0 (0.0) | | | 3 (2.9) |
| HR-/HER2- | | | | | | | | | | | | | |
| Yes | 94 (12.5) | 53 (31.0) | 9 (16.7) | 11 (18.6) | 54 (41.9) | 17 (37.0) | 19 (63.3) | 0 (0.0) | 1 (20.0) | 18 (45.0) | 104 (13.8) | 56 (30.6) | 34 (33.0) |
| No | 602 (80.2) | 108 (63.2) | 39 (72.2) | 46 (78.0) | 72 (55.8) | 28 (60.9) | 10 (33.3) | 2 (66.7) | 4 (80.0) | 21 (52.5) | 607 (80.5) | 121 (66.1) | 64 (62.1) |
| Unknown | 55 (7.3) | 10 (5.9) | 6 (11.1) | 2 (3.4) | 3 (2.3) | 1 (2.1) | 1 (3.34) | 1 (33.3) | 0 (0.0) | 1 (2.5) | 43 (5.3) | 6 (3.3) | 5 (4.9) |
| Hormone Status | | | | | | | | | | | | | |
| + | 594 (79.1) | 104 (60.8) | 32 (59.3) | 44 (74.6) | 44 (34.1) | 25 (54.4) | 10 (33.3) | 1 (33.3) | 4 (80.0) | 10 (25.0) | 587 (77.9) | 112 (61.2) | 34 (33.0) |
| - | 113 (15.1) | 63 (36.8) | 18 (33.3) | 14 (23.7) | 82 (63.6) | 20 (43.5) | 20 (66.7) | 2 (66.7) | 1 (20.0) | 27 (67.5) | 126 (16.7) | 67 (36.6) | 64 (62.1) |
| Unknown | 44 (5.9) | 4 (2.3) | 4 (7.4) | 1 (1.7) | 3 (2.3) | 1 (2.1) | 0 (0.0) | 0 (0.0) | 0 (0.0) | 3 (7.5) | 41 (5.4) | 4 (2.2) | 5 (4.9) |
| Histology | | | | | | | | | | | | | |
| Ductal | 514 (68.4) | 142 (83.0) | 44 (81.5) | 36 (61.0) | 119 (92.2) | 42 (91.3) | 24 (80.0) | 3 (100.0) | 4 (80.0) | 29 (76.3) | 504 (67.7) | 153 (83.6) | 91 (88.3) |
| Lobular | 162 (21.6) | 12 (7.0) | 4 (7.4) | 18 (30.5) | 1 (0.8) | 2 (4.3) | 2 (6.7) | 0 (0.0) | 1 (20.0) | 2 (5.3) | 166 (22.3) | 13 (7.1) | 0 (0.0) |
| Mucinous | 13 (1.7) | 2 (1.2) | 2 (3.7) | 0 (0.0) | 5 (3.9) | 0 (0.0) | 1 (3.3) | 0 (0.0) | 0 (0.0) | 0 (0.0) | 14 (1.9) | 1 (0.5) | 5 (4.9) |
| Other | 61 (8.1) | 14 (8.2) | 4 (7.4) | 5 (8.5) | 1 (0.8) | 1 (2.2) | 0 (0.0) | 0 (0.0) | 0 (0.0) | 7 (18.4) | 58 (7.8) | 15 (8.2) | 1 (1.0) |
| Unknown | 1 (0.1) | 1 (0.6) | 0 (0.0) | 0 (0.0) | 3 (2.3) | 1 (2.2) | 3 (10.0) | 0 (0.0) | 0 (0.0) | 0 (0.0) | 2 (0.3) | 1 (0.5) | 6 (5.8) |

Note: The number of TCGA samples with unassigned (ambiguous) value in ethnicity were 59 for whole exome sequence, and 5 for whole genome sequence.

## Supplementary Table 2. TCGA data comparison against SEER data

| | SEER | TCGA | Chi-square | *P* value |
|---|---|---|---|---|
| **Overall** | **50571** | **1098** | | |
| **HR&HER2** | | | 31.73 | <0.001 |
| HR+/HER2- | 36810 (72.7) | 684 (66.7) | | |
| Triple-negative | 6193 (12.2) | 178 (17.4) | | |
| HR+/HER2+ | 5240 (10.3) | 126 (12.3) | | |
| HR-/HER2+ | 2328 (4.6) | 38 (3.7) | | |
| **HR** | | | 12.97 | <0.001 |
| + | 42050 (83.2) | 824 (79.0) | | |
| - | 8521 (16.8) | 220 (21.0) | | |
| **HER2** | | | 0.45 | 0.504 |
| + | 7568 (15.0) | 168 (15.7) | | |
| - | 43003 (85.0) | 902 (84.3) | | |
| **Black** | **5268** | **176** | | |
| **HR&HER2** | | | 9.48 | 0.024 |
| HR+/HER2- | 3169 (60.2) | 86 (51.8) | | |
| Triple-negative | 1183 (22.5) | 54 (32.5) | | |
| HR+/HER2+ | 598 (11.4) | 18 (10.8) | | |
| HR-/HER2+ | 318 (6.0) | 8 (4.8) | | |
| **HR** | | | 6.18 | 0.013 |
| + | 3767 (71.5) | 108 (62.8) | | |
| - | 1501 (28.5) | 64 (37.2) | | |
| **HER2** | | | 0.5 | 0.478 |
| + | 916 (17.4) | 26 (15.3) | | |
| - | 4352 (82.6) | 144 (84.7) | | |
| **White** | **35985** | **798** | | |
| **HR&HER2** | | | 18.41 | <0.001 |
| HR+/HER2- | 27165 (75.5) | 530 (71.2) | | |
| Triple-negative | 3850 (10.7) | 104 (14.0) | | |
| HR+/HER2+ | 3532 (9.8) | 92 (12.4) | | |
| HR-/HER2+ | 1438 (4.0) | 18 (2.4) | | |
| **HR** | | | 1.85 | 0.174 |
| + | 30697 (85.3) | 629 (83.5) | | |
| - | 5288 (14.7) | 124 (16.5) | | |
| **HER2** | | | 0.31 | 0.579 |
| **+** | 4970 (13.8) | 113 (14.5) | | |
| **-** | 31015 (86.2) | 666 (85.5) | | |

Note: data extracted from Howlader N, et al. J Natl Cancer Inst (2014) 106(5):dju055.

**Supplementary Table 3. TCGA data comparison against SEER data, limited to non-Hispanic population**

| | SEER | TCGA | Chi-square | *P* value |
|---|---|---|---|---|
| **Overall** | **50571** | **883** | | |
| **HR&HER2** | | | 28.87 | <0.001 |
| HR+/HER2- | 36810 (72.7) | 545 (67.0) | | |
| Triple-negative | 6193 (12.2) | 150 (18.4) | | |
| HR+/HER2+ | 5240 (10.3) | 85 (10.4) | | |
| HR-/HER2+ | 2328 (4.6) | 34 (4.2) | | |
| **HR** | | | 18.57 | <0.001 |
| + | 42050 (83.2) | 644 (77.5) | | |
| - | 8521 (16.8) | 187 (22.5) | | |
| **HER2** | | | 0.24 | 0.628 |
| + | 7568 (15.0) | 123 (14.4) | | |
| - | 43003 (85.0) | 733 (85.6) | | |
| **Non-Hispanic Black** | **5268** | **158** | | |
| **HR&HER2** | | | 9.37 | 0.025 |
| HR+/HER2- | 3169 (60.2) | 77 (52.0) | | |
| Triple-negative | 1183 (22.5) | 49 (33.1) | | |
| HR+/HER2+ | 598 (11.4) | 15 (10.1) | | |
| HR-/HER2+ | 318 (6.0) | 7 (4.7) | | |
| **HR** | | | 6.14 | 0.013 |
| + | 3767 (71.5) | 96 (66.7) | | |
| - | 1501 (28.5) | 58 (23.3) | | |
| **HER2** | | | 0.88 | 0.349 |
| + | 916 (17.4) | 22 (14.5) | | |
| - | 4352 (82.6) | 130 (85.5) | | |
| **Non-Hispanic White** | **35985** | **631** | | |
| **HR&HER2** | | | 13.4 | 0.004 |
| HR+/HER2- | 27165 (75.5) | 420 (72.5) | | |
| Triple-negative | 3850 (10.7) | 87 (15.0) | | |
| HR+/HER2+ | 3532 (9.8) | 57 (9.8) | | |
| HR-/HER2+ | 1438 (4.0) | 15 (2.6) | | |
| **HR** | | | 3.74 | 0.053 |
| + | 30697 (85.3) | 484 (82.5) | | |
| - | 5288 (14.7) | 103 (17.5) | | |
| **HER2** | | | 1.26 | 0.262 |
| + | 4970 (13.8) | 75 (12.2) | | |
| - | 31015 (86.2) | 538 (87.8) | | |

Note: data extracted from Howlader N, et al. J Natl Cancer Inst (2014) 106(5):dju055.

## Supplementary Table 4. Comparison between TCGA Black and Nigeria

| | TCGA | Nigeria | Chi-square | *P* value |
|---|---|---|---|---|
| **Non-Hispanic Black** | **158** | **195** | | |
| **HR&HER2** | | | 51.81 | <0.001 |
| HR+/HER2- | 77 (52.0) | 37 (19.0) | | |
| Triple-negative | 49 (33.1) | 82 (42.1) | | |
| HR+/HER2+ | 15 (10.1) | 27 (13.9) | | |
| HR-/HER2+ | 7 (4.7) | 49 (13.9) | | |
| **HR** | | | 29.34 | <0.001 |
| + | 96 (66.7) | 66 (33.3) | | |
| - | 58 (23.3) | 132 (66.7) | | |
| **HER2** | | | 25.54 | <0.001 |
| + | 22 (14.5) | 78 (39.0) | | |
| - | 130 (85.5) | 122 (61.0) | | |

**Supplementary Table 5. Summary statistics for WES and WGS samples used for mutation signature analysis**

| | | Whole exome sequence (WES, N=500) | | | | | Whole genome sequence | WES&WGS |
|---|---|---|---|---|---|---|---|---|
| | | TCGA White | TCGA Black | TCGA Asian | TCGA Unassigned | Nigerians | (WGS, N=122)[a] | (N=59)[b] |
| Number | | 284 | 88 | 28 | 26 | 74 | 122 | 59 |
| Age | | 60.2±13.3 | 57.5±12.2 | 59.0±12.9 | 61.7±14.4 | 52.1±12.5 | 56.6±14.0 | 59.4±14.3 |
| ER | | | | | | | | |
| | + | 188 (66.2) | 42 (47.7) | 11 (39.3) | 18 (69.2) | 18 (24.3) | 48 (39.3) | 16 (27.1) |
| | - | 83 (29.2) | 44 (50.0) | 14 (50.0) | 8 (30.8) | 56 (75.6) | 73 (59.8) | 43 (72.9) |
| | Unknown | 13 (4.6) | 2 (2.3) | 3 (10.7) | 0 (0.0) | 0 (0.0) | 1 (0.9) | 0 (0.0) |
| PR | | | | | | | | |
| | + | 149 (52.5) | 32 (36.4) | 10 (35.7) | 10 (38.5) | 8 (10.8) | 35 (28.7) | 12 (20.3) |
| | - | 121 (42.6) | 54 (61.4) | 15 (53.6) | 16 (61.5) | 66 (89.2) | 84 (68.9) | 47 (79.7) |
| | Unknown | 14 (4.9) | 2 (2.3) | 3 (10.7) | 0 (0.0) | 0 (0.0) | 3 (2.5) | 0 (0.0) |
| HER2 | | | | | | | | |
| | + | 57 (20.1) | 11 (12.5) | 9 (32.1) | 5 (19.2) | 29 (39.2) | 34 (27.8) | 18 (30.5) |
| | - | 223 (78.5) | 75 (85.2) | 18 (64.3) | 21 (80.8) | 45 (60.7) | 85 (69.7) | 40 (67.8) |
| | Unknown | 4 (1.4) | 2 (2.3) | 1 (3.6) | 0 (0.0) | 0 (0.0) | 3 (2.5) | 1 (1.7) |
| | Equivocal | 0 (0.0) | 0 (0.0) | 0 (0.0) | 0 (0.0) | 0 (0.0) | 0 (0.0) | 0 (0.0) |
| HR-/HER2- | | | | | | | | |
| | Yes | 61 (21.5) | 37 (42.1) | 8 (28.6) | 7 (26.9) | 32 (43.2) | 54 (44.3) | 32 (54.2) |
| | No | 223 (78.5) | 51 (7.9) | 20 (71.4) | 19 (73.1) | 42 (56.7) | 65 (53.3) | 26 (44.1) |
| | Unknown | 0 (0.0) | 0 (0.0) | 0 (0.0) | 0 (0.0) | 0 (0.0) | 3 (2.5) | 1 (1.7) |
| Hormone Status | | | | | | | | |
| | + | 196 (69.0) | 43 (48.9) | 11 (39.3) | 18 (69.2) | 22 (29.7) | 50 (41.0) | 18 (30.5) |
| | - | 75 (26.4) | 43 (48.9) | 14 (50.0) | 8 (30.8) | 52 (70.3) | 68 (55.7) | 41 (69.5) |
| | Unknown | 13 (4.6) | 2 (2.3) | 3 (10.7) | 0 (0.0) | 0 (0.0) | 4 (3.3) | 0 (0.0) |

Note: [a] Three samples were excluded as outliers (due to their mutation number were either less than 100 for N009932 and N009975, or larger than 80,000 for TCGA-E2-A15K [White]).

[b] There were 24 TCGA Whites, 17 TCGA Blacks, 14 Nigerians, 2 TCGA Asians and 2 TCGA samples with unassigned (ambiguous) ethnicity.

**Supplementary Table 6. List of breast cancer driver genes**

| Copy Number | Short Variants |
|---|---|
| *AURKA* | *AKT1* |
| *CCND1* | *ARID1A* |
| *CCNE1* | *B2M* |
| *CDKN2A* | *BRCA1* |
| *EGFR* | *BRCA2* |
| *ERBB2* | *CASP8* |
| *FGFR1* | *CBFB* |
| *FOXO3* | *CDH1* |
| *IGF1R* | *CDKN1B* |
| *MAP2K4* | *CTCF* |
| *MCL1* | *CUL4B* |
| *MDM2* | *ERBB2* |
| *MDM4* | *FOXA1* |
| *MYC* | *FOXP1* |
| *MYCL* | *GATA3* |
| *RB1* | *GPS2* |
| *TBX2* | *HIST1H3B* |
| *TERT* | *HLA-DRB1* |
| *TP53* | *KDM6A* |
| | *KMT2A* |
| | *KMT2C* |
| | *KRAS* |
| | *MAP2K4* |
| | *MAP3K1* |
| | *MED23* |
| | *MLLT4* |
| | *MYB* |
| | *NCOR1* |
| | *NF1* |
| | *PIK3CA* |
| | *PIK3R1* |
| | *PLK2* |
| | *PTEN* |
| | *RAB40A* |
| | *RB1* |
| | *RUNX1* |
| | *SF3B1* |
| | *SPEN* |
| | *STAG2* |
| | *TBL1XR1* |
| | *TBX3* |
| | *TP53* |
| | *XBP1* |
| | *ZFP36L1* |

## Supplementary Table 7. Gene sets used to determine immune signatures

| Signature | Genes |
|---|---|
| T-cell | *CD3D, CD3E, CD3G, CD247, CD2, CD6* |
| B-cell | *CD19, CD20, CD79A, CD79B, CXCR5* |
| Cytotoxic cell | *PRF1, GZMA, GZMB, GZMH* |
| Macrophage | *CD68, CD163, CSF1R, FCGR1A, FCGR1B* |
| Fibroblast | *ACTA2, POSTN, FAP, COL1A1, COL1A2, PDGFRB* |
| IFN-gamma | *IFNG, CXCL9, CXCL10, CXCL11* |
| Type I interferon | *MX1, BST2, MX2, OAS1, OASL, OAS3, ISG15* |
| Proliferation | *MKI67, E2F1, MYBL2, BUB1, PLK1* |

**Supplementary Table 8. Validation of somatic mutations within novel significantly mutated genes**

| ID | Gene | Chrom | Pos | Ref | Alt | WGS_confirmed |
|---|---|---|---|---|---|---|
| L000077 | *PLK2* | 5 | 57755585 | C | T | Yes |
| N010829 | *PLK2* | 5 | 57754659 | C | G | Yes |
| TCGA-A8-A07I | *PLK2* | 5 | 57753373 | C | CTTTT | Yes |
| TCGA-A8-A07I | *PLK2* | 5 | 57753375 | TCAGGAGA | T | Yes |
| TCGA-B6-A0RE | *PLK2* | 5 | 57750416 | ACATCTT | A | Yes |
| TCGA-E2-A1LK | *KDM6A* | X | 44894228 | AAAGT | A | Yes |
| N010867 | *B2M* | 15 | 45003747 | G | T | Yes |
| TCGA-AR-A0TX | *B2M* | 15 | 45003751 | C | T | Yes |

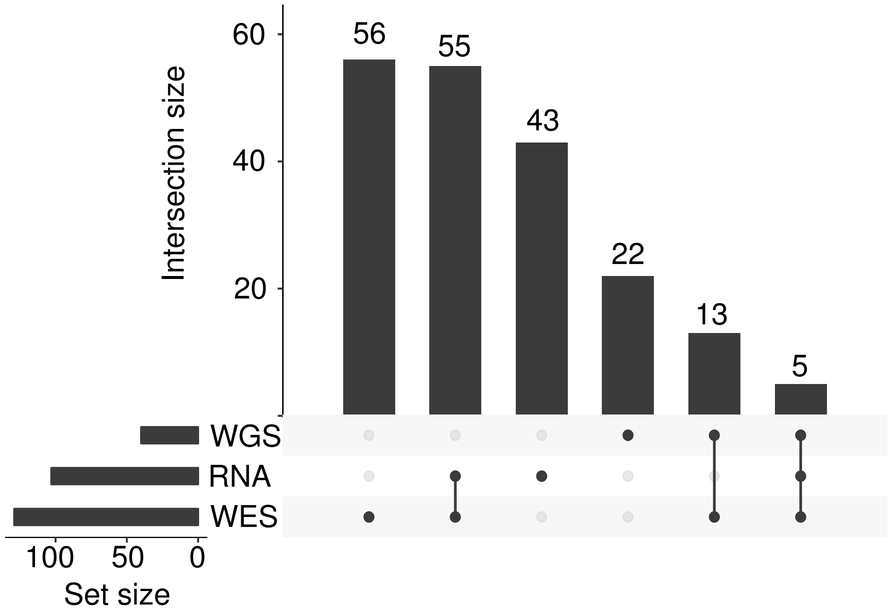


**Supplementary Figure 1**. The number of Nigerian samples with each NGS data type. WGS: Whole-genome sequencing, WES: whole-exome sequencing, RNA: mRNA sequencing.

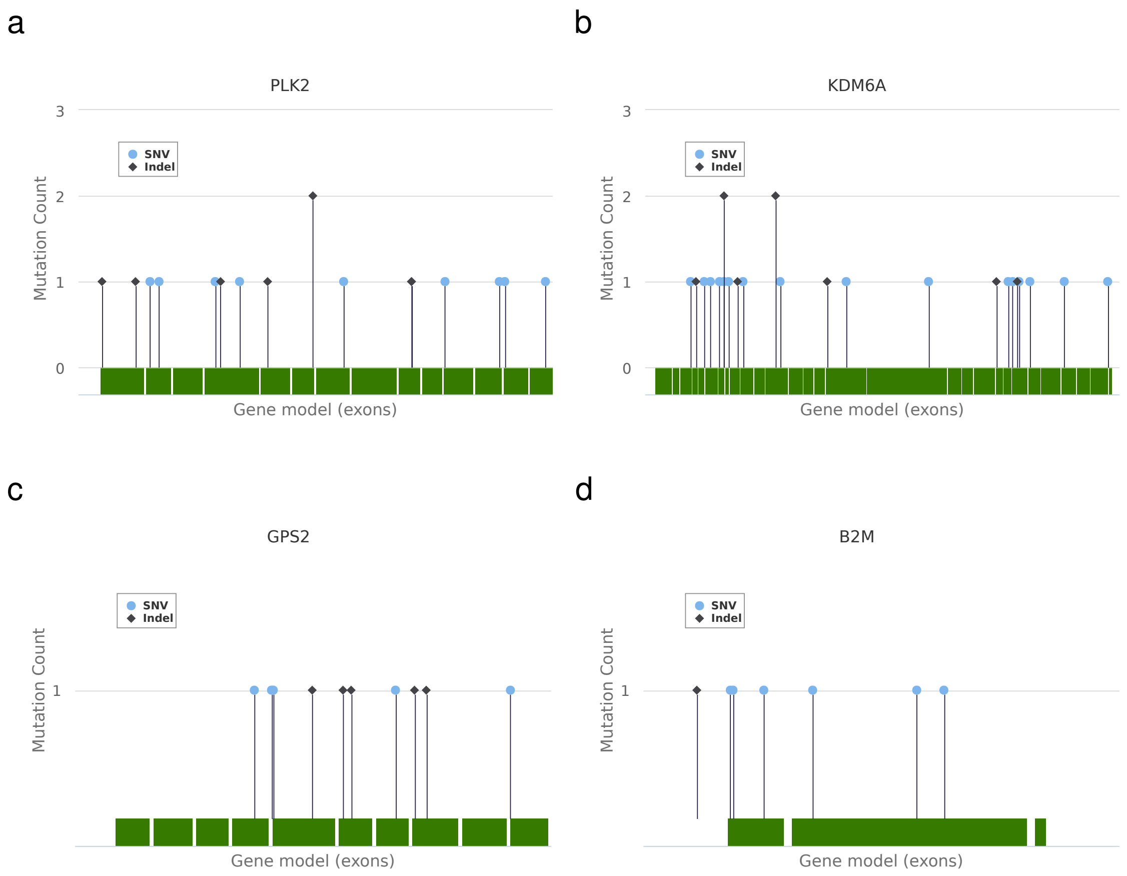


**Supplementary Figure 2**. Lollipop plots for novel significantly mutated breast cancer genes. Protein-altering SNVs and indels for **a** *PLK2*, **b** *KDM6A*, **c** *GPS2*, and **d** *B2M*. The start position of a deletion in *B2M* falls outside of the first exon; however, that deletion is represented in panel **d** since it spans part of the first exon.

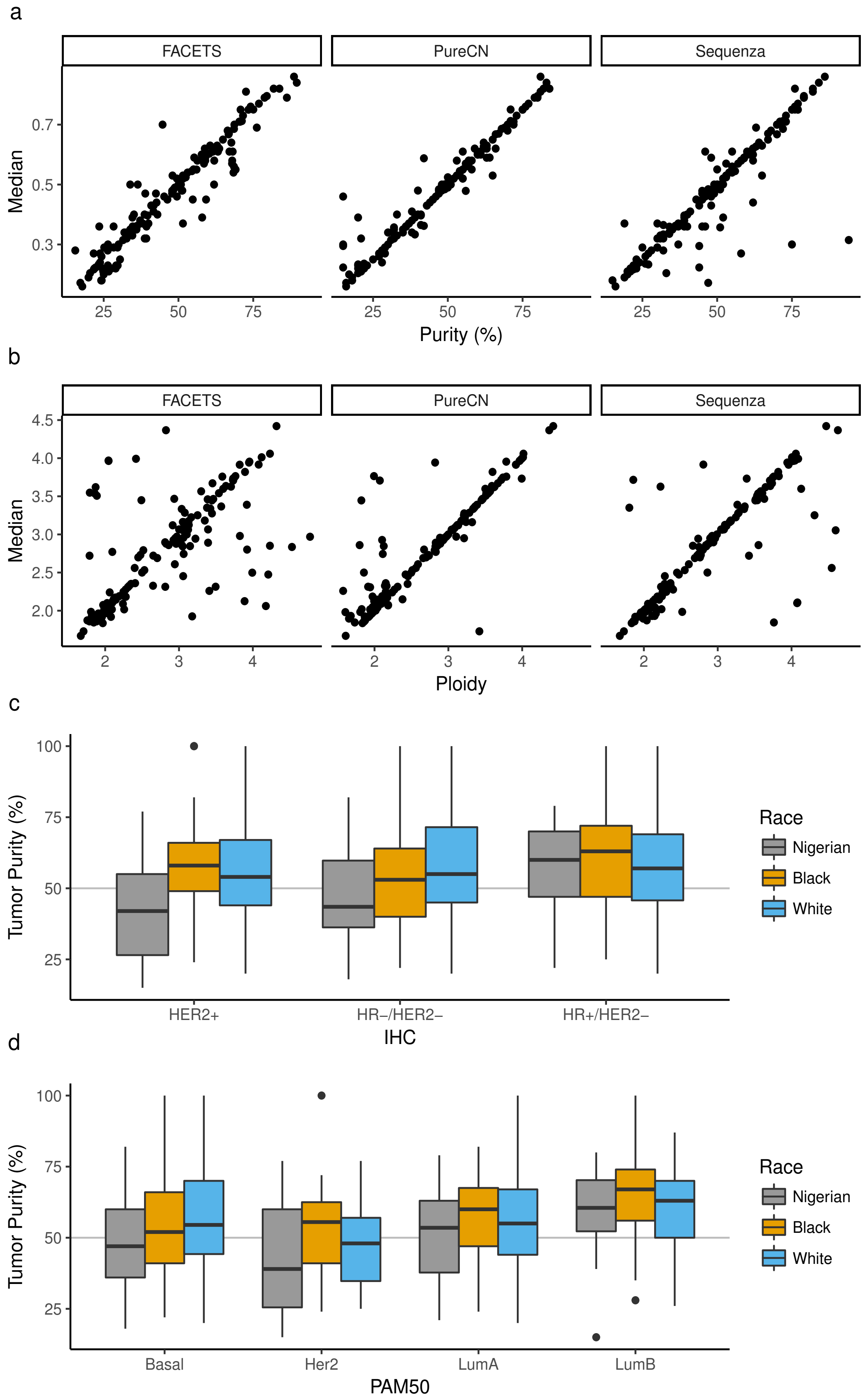


Pitt & Riester et al. Supplementary Figure 3

e f

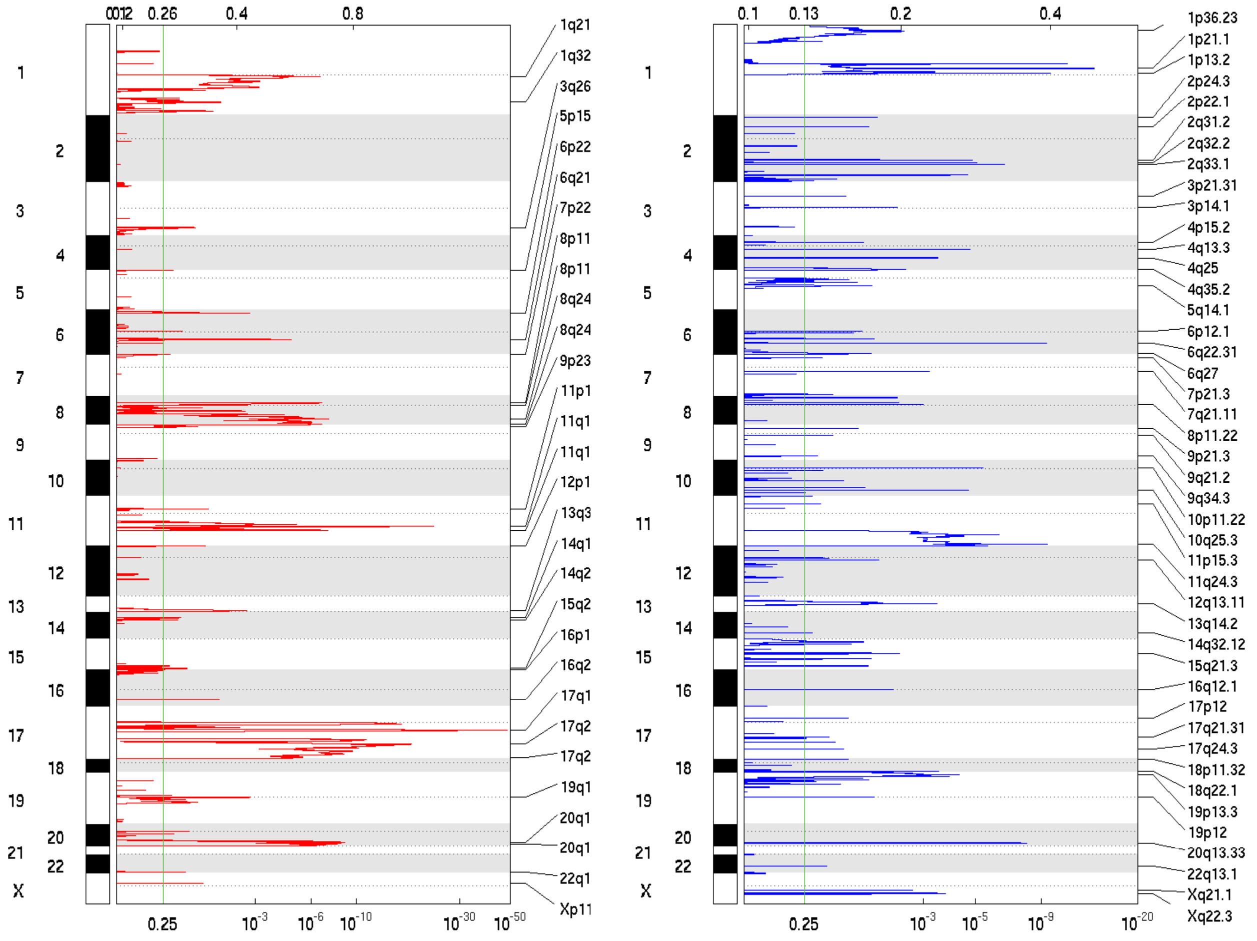


**Supplementary Figure 3**. Copy number calling in whole-exome sequencing data. Comparison of **a** purity and **b** ploidy estimates of the whole-exome samples obtained by 3 recently published tools. Shown are the tool estimates against the consensus (median) estimates of the 3 tools. Tumor purity comparison in **c** IHC and **d** PAM50 subtypes. TCGA is biased towards very high purity due to restrictive patient inclusion criteria. Shown are the curated purity estimates obtained by PureCN. Each box represents the upper and lower quartiles of the data, and the median is depicted with a horizontal line. Upper and lower whiskers extend to largest and smallest values within [1.5 x interquartile range], respectively. **e** Recurrent copy number gains and **f** losses as identified with GISTIC2.0. Q-values (green line indicates the default significance threshold of 0.25) are plotted on the x-axis against the genome position on the y-axis. GISTIC scores are plotted on the top x-axis, the corresponding Q-values on the bottom x-axis. Dotted lines show the centromere positions.

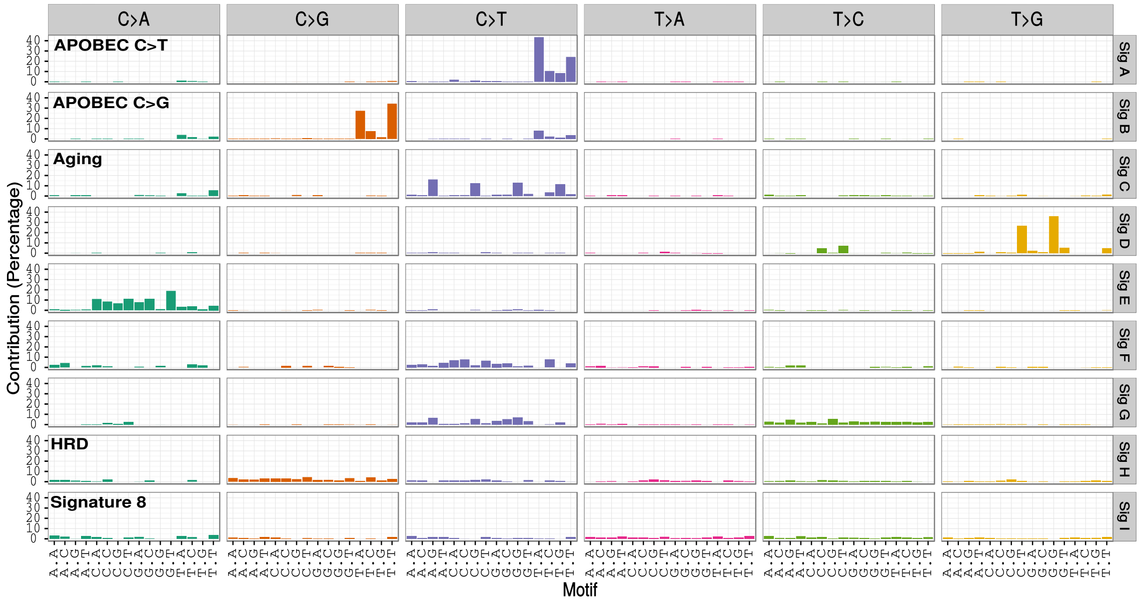


**Supplementary Figure 4**. Tri-nucleotide substitution patterns of nine inferred mutation signatures. Nine mutation signatures were jointly estimated from 500 exomes and 122 whole genomes using non-negative matrix factorization. All possible substitutions are represented within their tri-nucleotide context. Bars depict the percentage to which each tri-nucleotide substitution contributes to a given signature. APOBEC C>T, APOBEC C>G, aging, and HRD mutation signatures as well as COSMiC signature 8 are denoted.

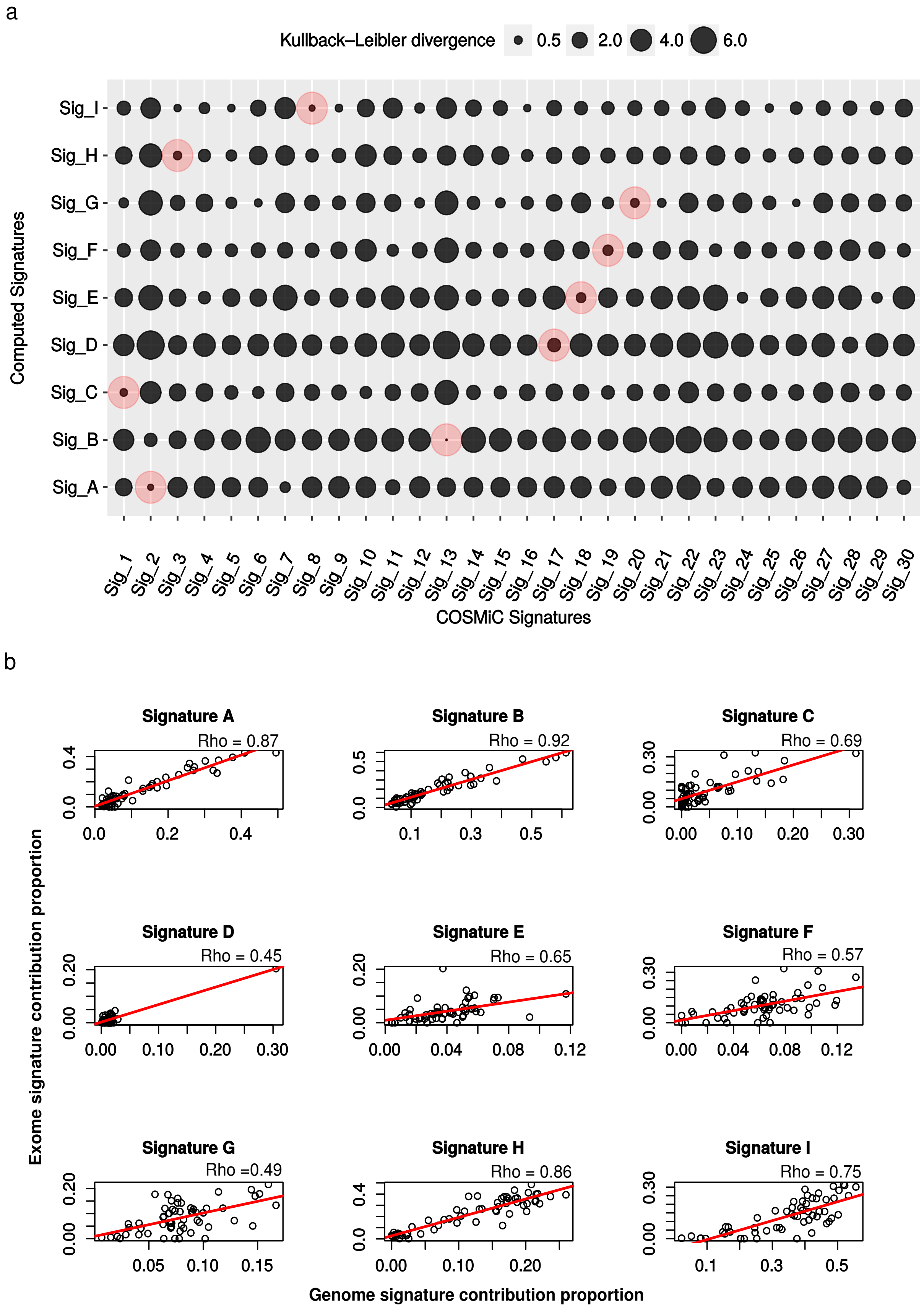


Pitt & Riester et al. Supplementary Figure 5

**Supplementary Figure 5**. Derived mutation signatures compared to COSMiC mutation signatures and correlation between WES and WGS signature contributions. **a** Kullback-Leibler divergence was calculated pairwise for derived and COSMiC mutation signatures. For each derived signature, the smallest divergence value — which indicates the most similar COSMiC signature — is denoted by a pink circle. **b** Scatterplots of nine mutation signature contributions between WES and WGS for 59 individuals. Spearman correlation was calculated for each signature with Rho depicted on each plot. The derived signature is denoted above each plot.

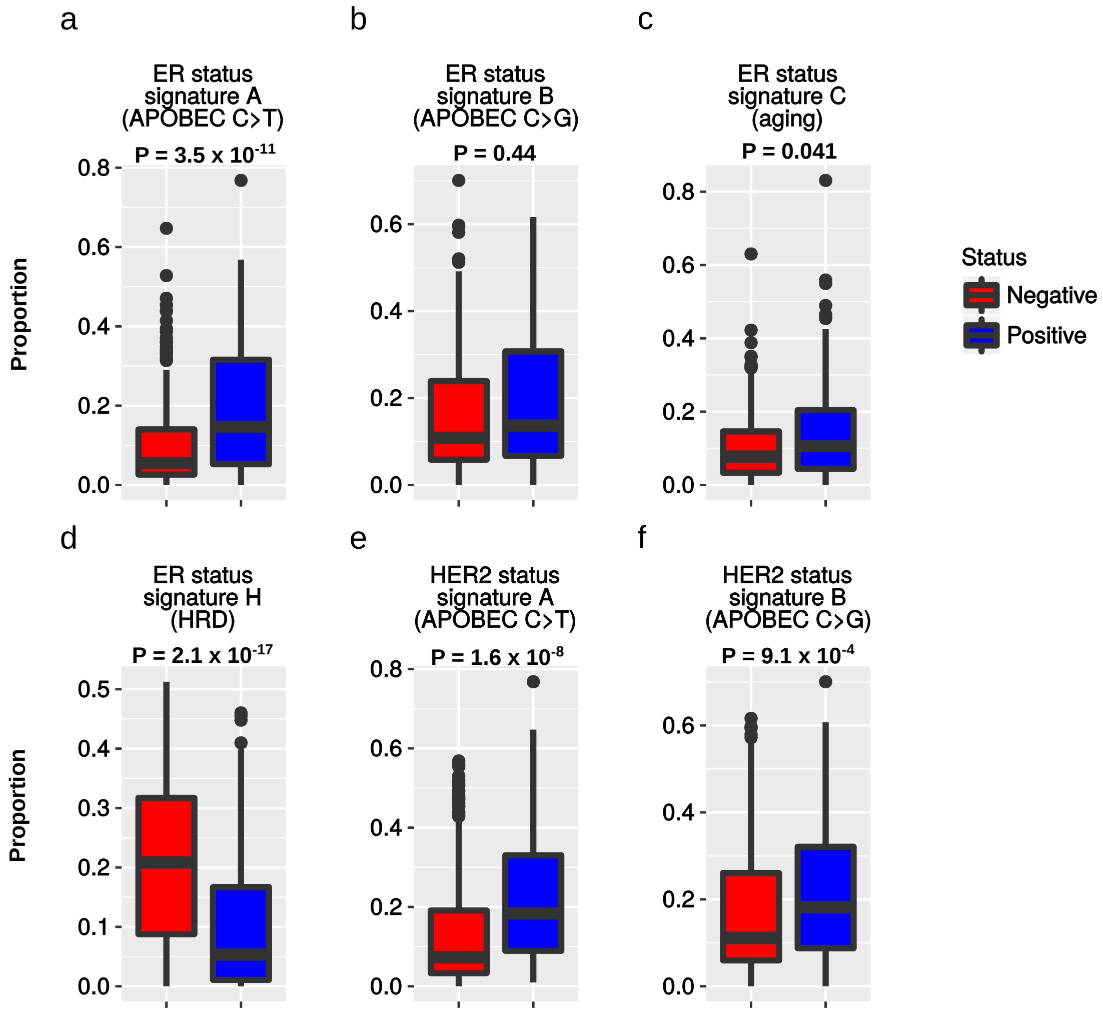


**Supplementary Figure 6**. Mutation signature contributions between tumors positive and negative for IHC markers. Boxplots represent **a** APOBEC C>T, **b** APOBEC C>G, **c** aging, and **d** HRD signatures partitioned by ER status (ER+ n = 277; ER- n = 205). Similarly, contributions from **e** APOBEC C>T and **f** APOBEC C>G signatures between HER2 positive (n = 111) and negative (n = 382) tumors. *P* values shown were calculated via Mann-Whitney U. Each box represents the upper and lower quartiles of the data, and the median is depicted with a horizontal line. Upper and lower whiskers extend to largest and smallest values within [1.5 x interquartile range], respectively

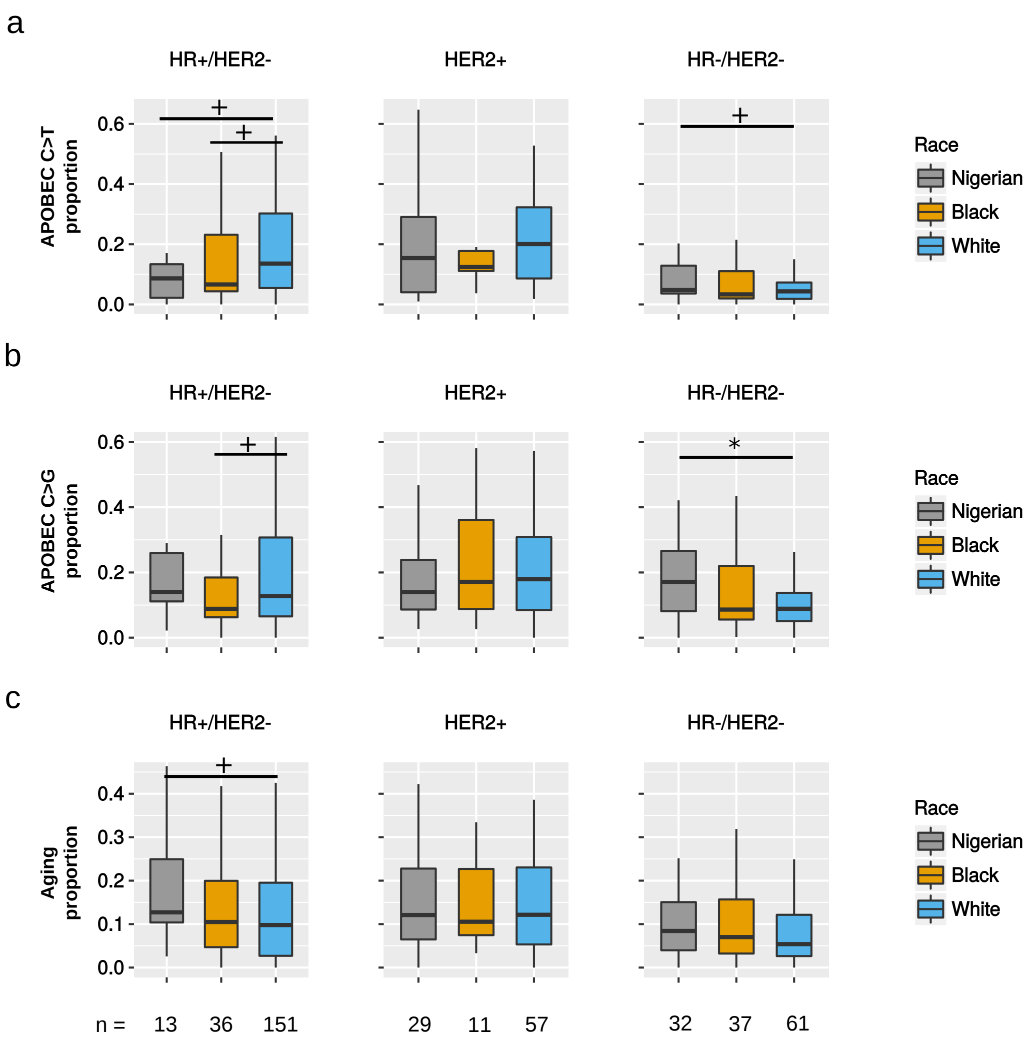


**Supplementary Figure 7**. The proportion of APOBEC C>T, APOBEC C>G, and aging signatures by race/ethnicity and IHC subtype using WES. Differences in **a** APOBEC C>T, **b** APOBEC C>G, and **c** aging signatures contributions by race/ethnicity within each IHC subtype were assessed using Kruskal-Wallis tests with post-hoc comparisons made via Dunn's test. Each box represents the upper and lower quartiles of the data, and the median is depicted with a horizontal line. Upper and lower whiskers extend to largest and smallest values within [1.5 x interquartile range], respectively.
* *P* values < 0.01; + *P* values ≤ 0.05.

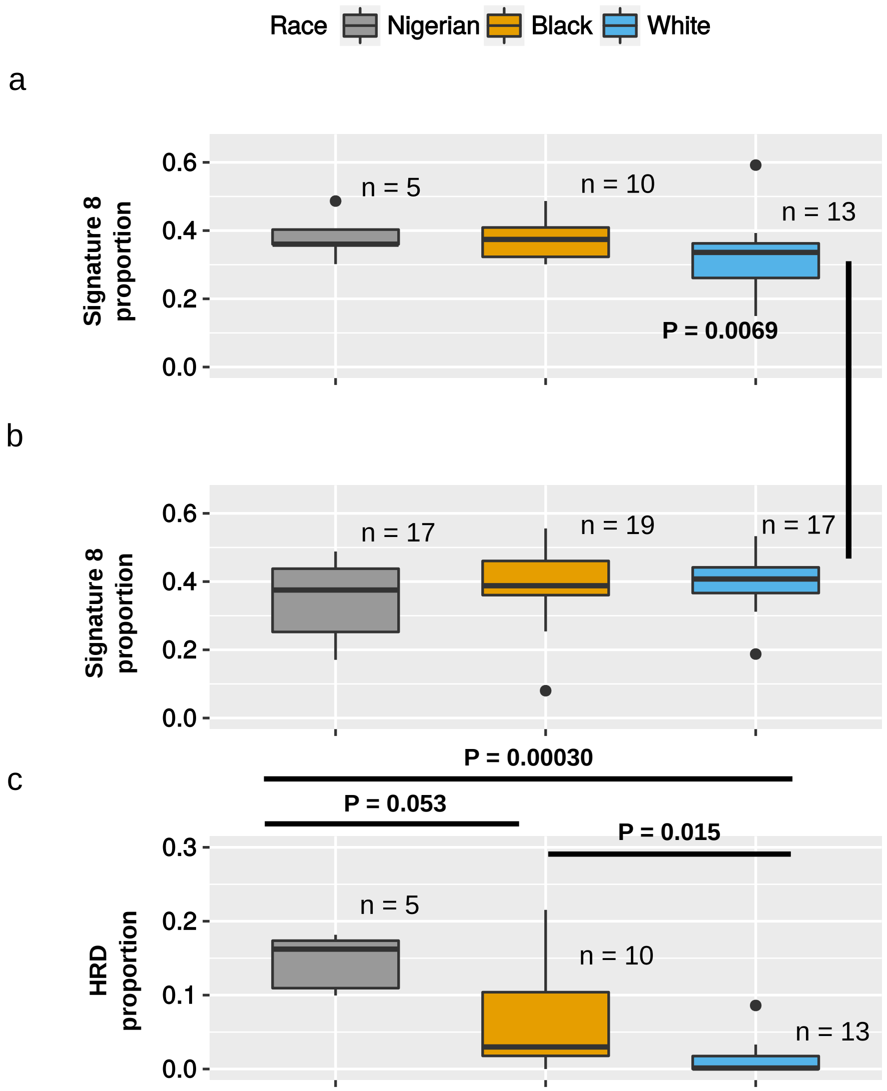


**Supplementary Figure 8**. Mutation signature contributions by race/ethnicity using WGS. Boxplots of WGS signature 8 contributions for **a** HR+/HER2- and **b** HR-/HER2- malignancies. **c** The proportion of HRD signature within HR+/HER2- malignancies. Racial/ethnic differences across subtypes were assessed using Kruskal-Wallis tests followed by *post-hoc* comparisons with Dunn's test. Within a race/ethnicity, tests across HR+/HER2- and HR-/HER2- (i.e. White in panels **a** and **b**) were performed with a Mann-Whitney U. *P* values < 0.05 are provided. Each box represents the upper and lower quartiles of the data, and the median is depicted with a horizontal line. Upper and lower whiskers extend to largest and smallest values within [1.5 x interquartile range], respectively.

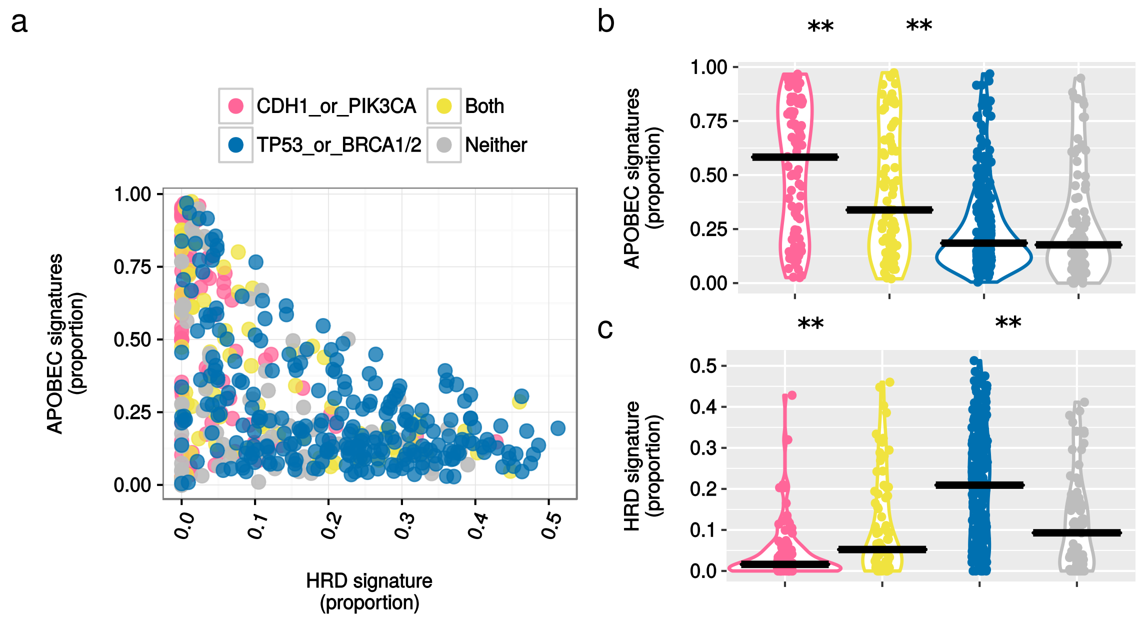


**Supplementary Figure 9**. Driver genes associate with APOBEC and HRD signature balance across all breast cancer IHC subtypes. **a** For each malignancy, the proportion of APOBEC signatures (sum of APOBEC C>T and C>G) by the proportion of HRD is shown. Each patient is colored based on harboring a *TP53* or *BRCA1/2* (including germline) mutation (blue), a *CDH1* or *PIK3CA* mutation (pink), mutations from both aforementioned categories (yellow), or mutations in neither of the aforementioned categories (grey). These values are decomposed into violin plots for **b** APOBEC and **c** HRD signatures, respectively. Horizontal black bars represent the median contribution proportion for each group. Between group comparisons were made using a Kruskal-Wallis test followed by Dunn's test.
** indicates groups that are significantly different ($P < 0.05$) from all three other categories.

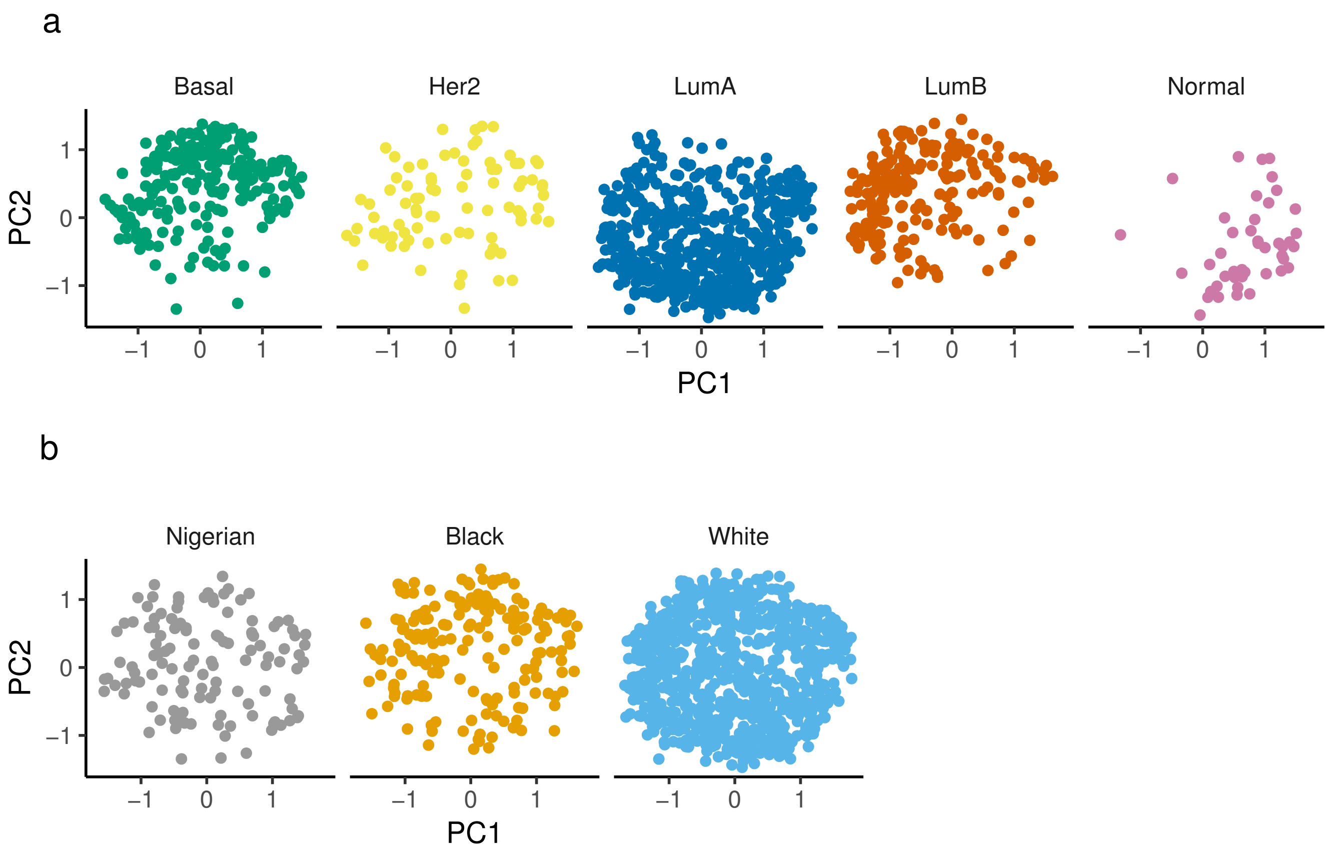


**Supplementary Figure 10**. Principal Component Analysis (PCA) of the immune signature scores by **a** PAM50 subtype, and **b** by race/ethnicity. Each dot represents a sample. Most variance is explained by subtype, not race. PC1 is most strongly associated with B-Cell and Cytotoxic cell signatures (and tumor purity), PC2 is highly correlated with the Fibroblast signature.

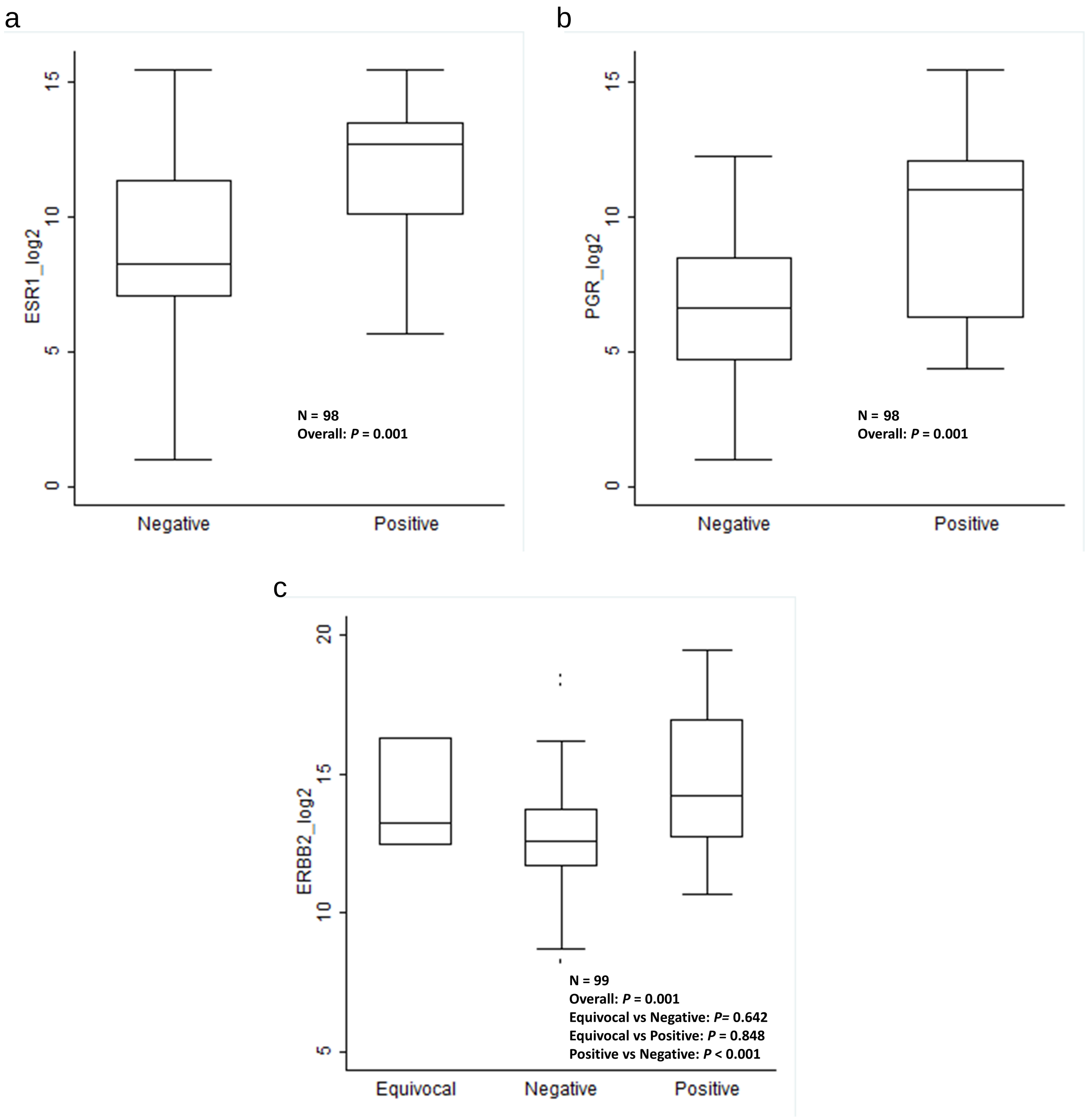


**Supplementary Figure 11**. Concordance amongst gene expression and immunohistochemistry calls. **a** *ESR1* expression by estrogen receptor (ER) status, **b** *PGR* expression by progesterone receptor (PR) status, and **c** *ERBB2* expression by HER2 status. Comparisons between groups were made using two-sample *t*-tests. Each box represents the upper and lower quartiles of the data, and the median is depicted with a horizontal line. Upper and lower whiskers extend to largest and smallest values within [1.5 x interquartile range], respectively.

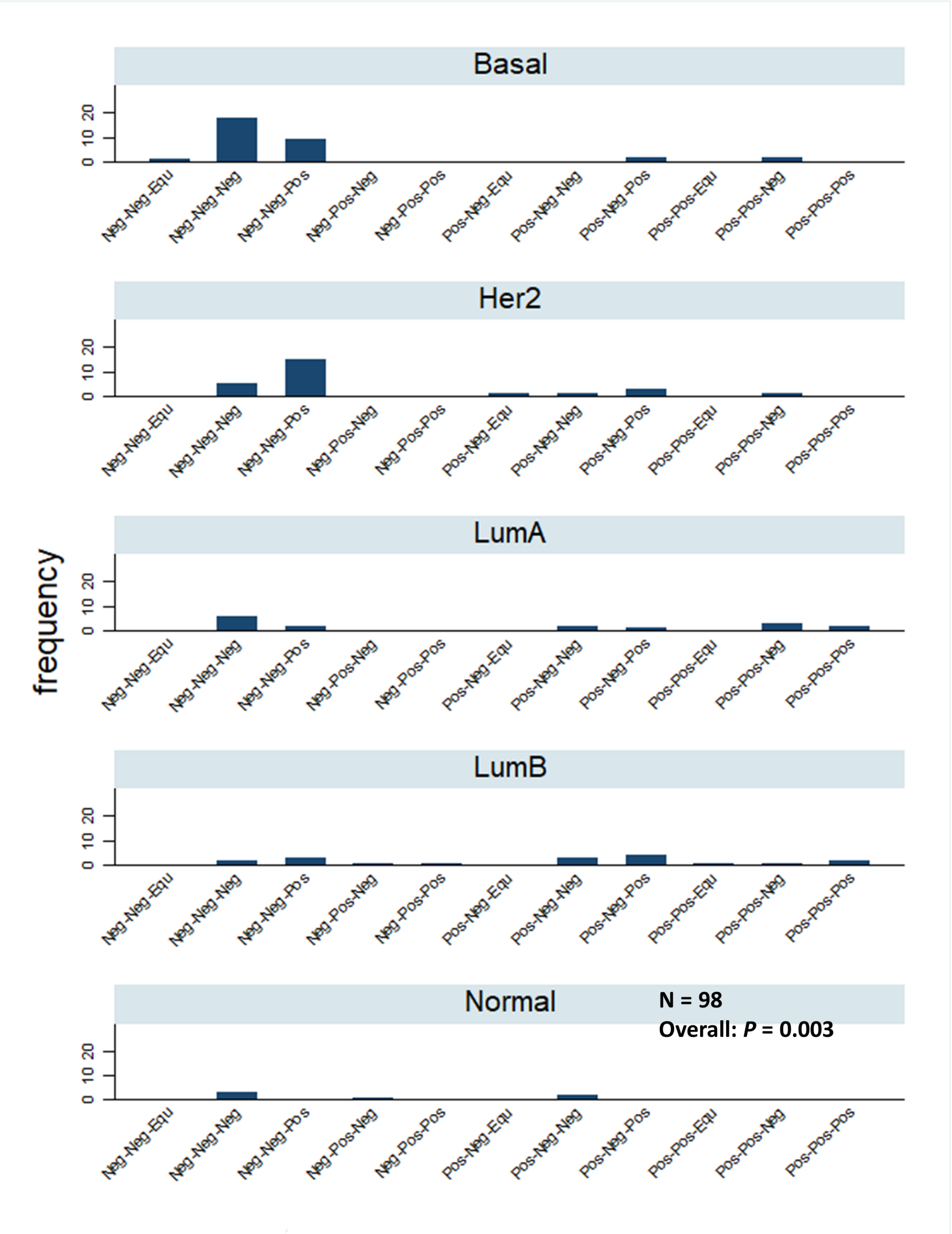


**Supplementary Figure 12**. Comparison of immunohistochemistry (IHC) subtypes and PAM50 classifications. A chi square test was used to assess if IHC subtypes were non-randomly distributed across PAM50 groups.

# Supplementary References


1. Riester, M. *et al.* PureCN: copy number calling and SNV classification using targeted short read sequencing. *Source Code Biol. Med.* **11,** 13 (2016).
2. Shen, R. & Seshan, V. E. FACETS: allele-specific copy number and clonal heterogeneity analysis tool for high-throughput DNA sequencing. *Nucleic Acids Res.* **44,** e131 (2016).
3. Favero, F. *et al.* Sequenza: allele-specific copy number and mutation profiles from tumor sequencing data. *Ann. Oncol.* **26,** 64–70 (2015).
4. Mermel, C. H. *et al.* GISTIC2.0 facilitates sensitive and confident localization of the targets of focal somatic copy-number alteration in human cancers. *Genome Biol.* **12,** R41 (2011).
5. Van Loo, P. *et al.* Allele-specific copy number analysis of tumors. *Proc. Natl. Acad. Sci. U. S. A.* **107,** 16910–16915 (2010).
6. Wang, K. *et al.* PennCNV: an integrated hidden Markov model designed for high-resolution copy number variation detection in whole-genome SNP genotyping data. *Genome Res.* **17,** 1665–1674 (2007).
7. Gehring, J. S., Fischer, B., Lawrence, M. & Huber, W. SomaticSignatures: inferring mutational signatures from single-nucleotide variants. *Bioinformatics* **31,** 3673–3675 (2015).
8. Kim, J. *et al.* Somatic ERCC2 mutations are associated with a distinct genomic signature in urothelial tumors. *Nat. Genet.* **48,** 600–606 (2016).
