# Supplementary material for: Characterization of Nigerian breast cancer reveals prevalent homologous recombination deficiency and aggressive molecular features
Source: Nat Commun. 2018 Oct 16;9:4181. doi: 10.1038/s41467-018-06616-0 (PMC6191428; doi:10.1038/s41467-018-06616-0)
Supplement: Supplementary file 3 — Description of Additional Supplementary Files [file 41467_2018_6616_MOESM3_ESM.pdf]

Description of Additional Supplementary Files

File Name: Supplementary Data 1
Description: Identifiers and relevant IHC data for all samples included in WES, WGS, RNA-seq, and array-based copy number analyses (ASCAT).

File Name: Supplementary Data 2
Description: Coverage information for Nigerian WES and WGS tumor-normal pairs.
